# Photoinduced Electron Transfer (PET) as Key to Accelerate the Cycloreversion Reaction of Arylquadricyclanes

**DOI:** 10.1002/chem.202502413

**Published:** 2025-09-29

**Authors:** Julian Felix Maria Hebborn, Heiko Ihmels, Marco Löcker, Thomas Paululat, Robin Schulte

**Affiliations:** ^1^ Department of Chemistry‐Biology Research Center of Micro‐ and Nanochemistry and (Bio‐)Technology (*Cμ*), University of Siegen, Adolf‐Reichwein‐Str. 2 Siegen 57068 Germany

**Keywords:** molecular solar thermal energy storage (MOST), norbornadiene, photocatalysis, photochromism, radical ions

## Abstract

The development of methods for the utilization of sustainable energy resources is an important scientific challenge. In one approach, the conversion and storage of solar light energy as chemical energy in a photoreaction has been established, namely as molecular solar thermal energy storage (MOST). In particular, the norbornadiene‐quadricyclane system offers favorable photochemical and physicochemical properties for this purpose. However, the chemical reaction to release the stored energy, i.e., the cycloreversion of the quadricyclane, still requires improvement. In this context, we demonstrate that a fast and controlled cycloreversion of 2‐(5‐(1‐methoxynaphthyl))quadricyclane can be accomplished in a photoinduced electron transfer (PET) reaction. Specifically, we identified 9‐mesityl‐10‐methylacridinium, 9,10‐dicyanoanthracene, and 9‐nitrobenzo[*b*]quinolizinium as suitable catalysts that initiate the fast cycloreversion of the quadricyclane to the norbornadiene with visible light (420 nm) and with low catalyst loading. At the same time, there is no significant loss of favorable MOST properties, namely half‐life and energy storage density. Thus, this method provides an efficient, complementary tool for the targeted cycloreversion of quadricyclane with high temporal and local control, as required for MOST applications.

## Graphical abstract

It is demonstrated that the photoinduced cycloreversion of a methoxynaphthylquadricyclane in the presence of suitable photocatalysts gives quantitatively the corresponding norbornadiene. As this photochromic norbornadiene‐quadricyclane cycle has favorable properties for light energy conversion, this method provides a useful tool for the targeted release of stored energy with high temporal and local control.

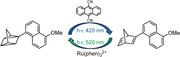

## 1 Introduction

**Scheme 1:**
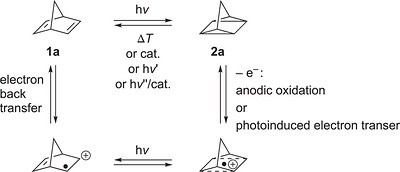
Reversible photoinduced isomerization of norbornadiene (**1a**) to quadricyclane (**2a**), and different triggers for the back reaction.

Global energy demand is rising steadily because of the growing global population and the expansion of large‐scale industrial processes.^[^ ^1^ ^]^ At the same time, there is an urgent need to reduce emissions from fossil sources^[^ ^2^ ^]^ used to supply energy. Therefore, the development and exploitation of sustainable energy resources is a key task of scientific research.^[^ ^1^, ^3^ ^]^ In this context, the use of solar power has attracted particular interest because sunlight is virtually an inexhaustible source of energy.^[^ ^4^ ^]^ At the same time, however, the direct use of sunlight is dependent on the weather conditions and daytime, so that storage systems are required for an efficient and flexible harvesting and conversion of this energy.^[^ ^5^ ^]^ From a chemical perspective, such storage of light energy as chemical energy may be accomplished by means of a reversible photochemical reaction.^[^ ^6^, ^7^ ^]^ To use such a photochromic reaction for this purpose, a metastable photoproduct with much higher energy has to be formed. In turn, the stored energy is released in the form of heat, i.e., as reaction enthalpy, once the back reaction is triggered, ideally in a controlled way with heat, light, or a catalyst. This main principle has been established with the molecular solar thermal energy storage (MOST) systems, which employ suitable photochromic reactions for light energy storage.^[^ ^7^, ^8^, ^9^ ^]^ In particular, the photochromic cycle between norbornadiene (**1a**) and quadricyclane (**2a**) has attracted special attention as a promising system for light energy conversion and storage because of its high energy storage density (Scheme 1).^[^ ^10^ ^]^ As a drawback, however, the parent system has critical photochemical parameters, such as a low quantum yield (*Φ* = 5%) and a negligible overlap of the absorption with the solar emission spectrum, especially in the visible range. Nevertheless, in several approaches toward optimization, some of these issues have been solved by chemical modification of the norbornadiene scaffold or the use of photocatalysts to achieve more favorable quantum yields and red‐shifted absorption.^[^ ^11^, ^12^ ^]^ But while the latter properties have been significantly improved over the years, the controlled release of the stored energy with the cycloreversion reaction still needs some special attention. This back reaction may be triggered with different physical or chemical stimuli, for example, heat,^[^ ^13^, ^14^ ^]^ voltage,^[^ ^15^, ^16^ ^]^ acids,^[^ ^17^ ^]^ transition metal catalysts,^[^ ^18^ ^]^ or radical cations^[^ ^19^, ^20^ ^]^ (Scheme 1). But with regards to application, a light‐induced cycloreversion appears to be particularly practicable because it can be performed with high local and temporal control. In fact, the quadricyclane can be transformed back to norbornadiene upon direct irradiation.^[^ ^21^, ^22^ ^]^ However, this photoreaction requires high‐energy excitation and is thus accompanied by decomposition reactions.^[^ ^22^ ^]^ Quadricyclane (**2a**) has also been converted into norbornadiene by a photoinduced electron transfer (PET) reaction with suitable excited‐state electron acceptors, such as chloranil or methylene blue, which enables excitation with lower‐energy light (Scheme 1).^[^ ^23^, ^24^ ^]^ The mechanism has been proposed to proceed through the formation of the corresponding radical cations (Scheme 1).^[^ ^24^, ^25^, ^26^ ^]^ In a similar approach, covalently linked conjugates of a norbornadiene unit and a suitable electron acceptor functionality with potential photocatalyst activity, such as fullerene (C_60_),^[^ ^27^ ^]^ perylene diimine,^[^ ^28^ ^]^ and naphthalene diimide,^[^ ^29^ ^]^ have been investigated recently. Hence, the cycloreversion of the corresponding quadricyclanes is initiated upon selective excitation of the attached photocatalyst. The mechanism has been described as an oxidative cycloreversion induced by the charge separation resulting from an intramolecular PET process.^[^ ^27^, ^28^, ^29^ ^]^ Even though this system provides efficient and controllable energy release, it has a relatively low energy density because of the large molecular mass.^[^ ^28^ ^]^ Complementary to the light‐induced cycloreversion through formation of radical ions, the reaction may also be initiated electrochemically, i.e., by anodic oxidation (Scheme 1),^[^ ^16^ ^]^ in some cases even with reversibility efficiency of >99% over 1000 cycles.^[^ ^15^ ^]^

In this context, we aimed at the identification of PET‐based cycloreversion reactions of appropriately designed quadricyclane derivatives. Specifically, we proposed that monoaryl‐substituted norbornadienes^[^ ^13^, ^30^ ^]^ offer the advantage to adjust the propensity of the compound to be involved in a PET process with suitable catalysts by attachment of donor or acceptor substituents to the arene unit, while still providing the required MOST properties.^[^ ^11^, ^13^ ^]^ Most importantly, this design principle may provide photoreactions with low‐energy light, if the PET sensitizers absorb in the visible region. As preliminary proof of concept, we demonstrate herein that, indeed, the cycloreversion of the methoxynaphthylquadricyclane **2d** upon irradiation in the presence of excited‐state acceptors gives quantitatively the norbornadiene **1d**.

## 2 Results and Discussion

**Scheme 2:**
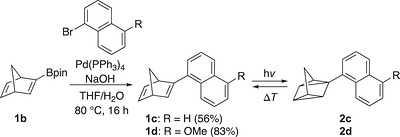
Synthesis and photochromic reactions of naphthyl‐substituted norbornadienes **1c** and **1d** (pin = pinacolyl).

According to the known protocol for the synthesis of the parent naphthylnorbornadiene **1c** the derivative **1d** was synthesized by Suzuki‐Miyaura coupling reaction of the borononorbornadiene **1b** ^[^ ^30a^ ^]^ with 1‐bromo‐5‐methoxynaphthalene in 83% yield (Scheme 2).^[^ ^13^, ^30^ ^]^ The product **1d** was identified and characterized by NMR spectroscopy (^1^H, ^13^C, COSY, HSQC, HMBC), melting point, and elemental analysis. The norbornadiene **1d** is well soluble in polar and nonpolar solvents, but it is poorly soluble in protic, polar solvents, like MeOH, and insoluble in water.

**Figure 1:**
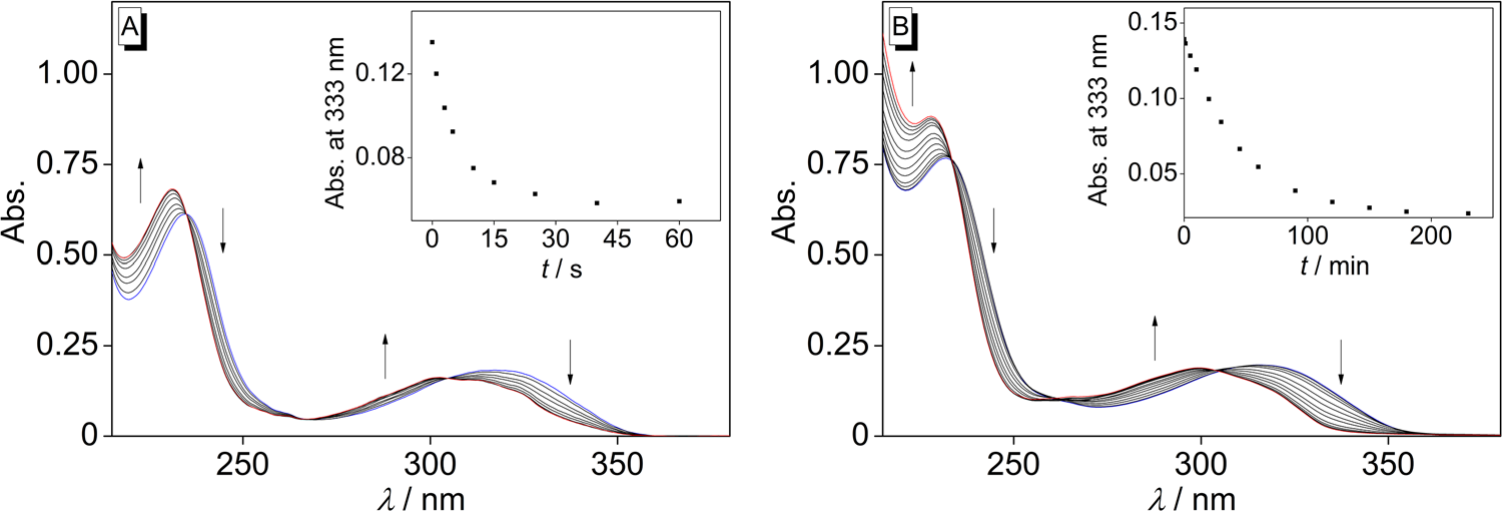
Photometric monitoring of the direct irradiation of **1d** in MeCN (*c* = 20 µM) at 315 nm, A) and of the photosensitized reaction at 520 nm in the presence of Ru(phen)_3_(PF_6_)_2_ (1 molar equiv., B) Insets: plot of absorption at 333 nm versus reaction time.

**Scheme 3:**
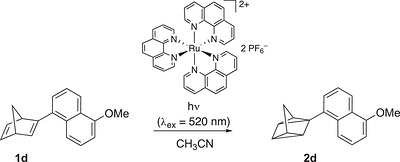
Photoisomerization of norbornadiene **1d** to quadricyclane **2d** upon photosensitization with Ru(phen)_3_(PF_6_)_2_ (*λ*_ex_ = 520 nm).

The absorption spectrum of **1d** is basically the same in cyclohexane, MeCN, EtOH, CHCl_3_, EtOAc, and benzene, and exhibits two main bands with maxima at 234 nm and 320 nm, the latter assigned to the naphthalene chromophore. The long‐wavelength absorption onset is located at 359 nm. The norbornadiene **1d** has similar absorption properties as the parent 1‐norbornadienylnaphthalene (**1c**),^[^ ^13^ ^]^ with slightly red‐shifted absorption maxima and absorption onset caused by the auxochromic effect of the methoxy group.^[^ ^31^ ^]^ The [2 + 2] photocycloaddition of the norbornadiene **1d** was monitored in MeCN by absorption spectroscopy. Upon irradiation of **1d** at *λ*_ex_ = 315 nm, new absorption maxima formed at 300 nm and 228 nm, and isosbestic points developed during the photoreaction (Figure 1). In comparison with **1c**, the quantum yield of the photoisomerization of **1d** (*Φ* = 21%) is lower, which may indicate additional deactivation pathways of the excited state, likely by rotation of the methoxy group. To gain more information about the structure of the photoproduct, possible byproducts, and the photostationary state (PSS), the sample was irradiated in MeCN‐*d*_3_, and the reaction was monitored at different time intervals by ^1^H NMR spectroscopy. Upon irradiation at *λ*_ex_ = 315 nm, the norbornadiene **1d** was predominantly converted to the corresponding quadricyclane **2d** with a **1d**/**2d** ratio of 72:28 in the PSS. In addition, the photoisomerization reaction of **1d** was also induced by irradiation with *λ*_ex_ = 520 nm in the presence of the triplet sensitizer Ru(phen)_3_(PF_6_)_2_,^[^ ^11^ ^]^ as monitored directly by absorption spectroscopy and in‐situ ^1^H NMR spectroscopy (Scheme 3, Figure 1).^[^ ^32^ ^]^ The photometric analysis revealed similar changes of the absorption spectrum during photosensitization as the ones observed upon direct irradiation. However, the photosensitized reaction was slower (approx. 3.8 hours) than the one upon direct irradiation (approx. 40 s), presumably because the low concentration (*c* = 20 µM) is not favorable for this bimolecular reaction. Furthermore, the direct NMR‐spectroscopic analysis of the photosensitized reaction showed a quantitative conversion of the norbornadiene **1d** to the respective quadricyclane **2d** (5 minutes, *c* = 60 mM) without detectable byproducts. Thus, the sensitized photoreaction of **1d** is a viable and mild method for the synthesis of quadricyclane **2d** with visible light.

The kinetic analysis of the thermal cycloreversion to the norbornadiene **1d** at 80 °C and extrapolation of the data showed a half‐life, *t*_1/2,_ of the quadricyclane **2d** of 75 d at 25 °C. Further analysis revealed a free activation enthalpy of 113 kJ mol^−1^ for the cycloreversion of **2d**. In addition, differential‐scanning calorimetry (DSC) analysis indicated an energy density of the quadricyclane **2d** of 329 kJ/kg, which is slightly lower than the one of 1‐quadricyclylnaphthalene (**2c**) because of the higher molecular mass of **2d**.^[^ ^13^ ^]^ However, this value still lies above 300 kJ/kg, that is, the commonly suggested threshold energy density to be considered for MOST applications.^[^ ^9^, ^33^ ^]^

**Figure 2:**
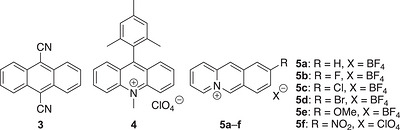
Structures of the photocatalysts 9,10‐dicyanoanthracene (**3**), 9‐mesityl‐10‐acridinium perchlorate (**4**), and benzo[*b*]quinolizinium salts **5a**–**f**.

**Figure 3:**
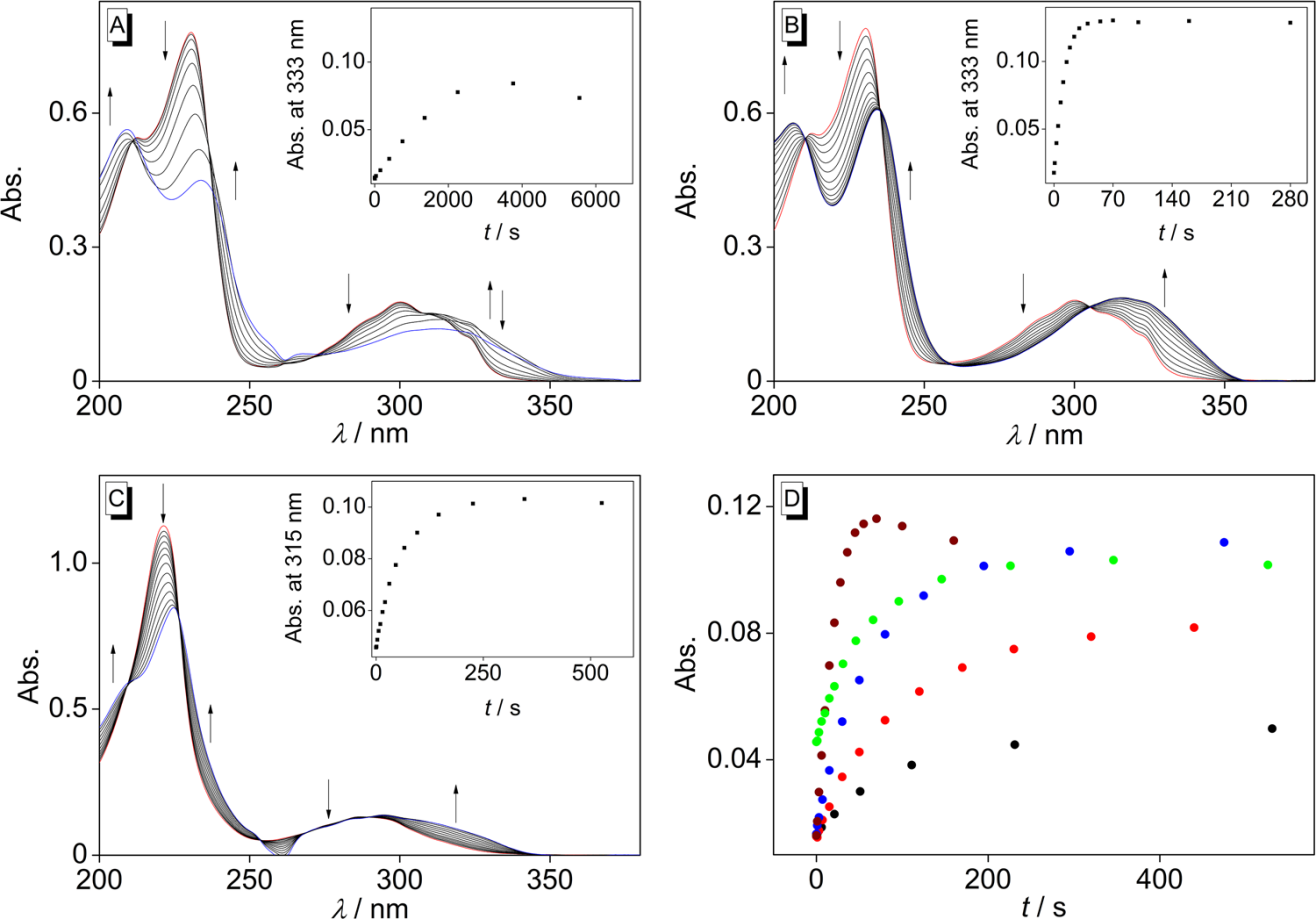
A,B: Photometric monitoring of the photoinduced cycloreversion of **2d** (*c* = 20 µM) in MeCN in the presence of 9,10‐dicyanoanthracene (**3**) (A, 10 mol%) and 9‐nitrobenzo[*b*]quinolizinium perchlorate (**5f**) (B, 7.5 mol%) at *λ*_ex_ = 420 nm. Insets: plot of absorption at 333 nm versus reaction time for the cycloreversion of **2d** with **3** and **5f** respectively. C: photometric monitoring of the photoinduced cycloreversion of **2c** (*c* = 20 µM) in MeCN in the presence of **4** (5 mol%) at *λ*_ex_ = 420 nm. Inset: plot of absorption at 315 nm versus reaction time for the cycloreversion of **2c** with **4**. D: plot of the absorption versus reaction time of **2d** with varying amounts of catalyst **4** (black: 0.5%, red: 1%, blue: 2%, brown: 5%) and of **2c** with catalyst **4** (green: 5%).

The photoinduced cycloreversion of the quadricyclane **2d** to the norbornadiene **1d** in the presence of selected photocatalysts (Figure 2) was investigated by absorption spectroscopy and in‐situ NMR spectroscopy (Figure 3). For the photometric analysis, a solution of **2d** and 9,10‐dicyanoanthracene (**3**) was irradiated with *λ*_ex_ = 420 nm. The photometric analysis showed the formation of the norbornadiene **1d** by the evolution of the characteristic absorption bands (Figure 3). Nevertheless, incomplete conversion and/or decomposition were also indicated by an additional decline in absorption (after approx. 60 min). In another approach, 9‐mesityl‐10‐methylacridinium (**4**) was employed as photocatalyst for the cycloreversion. These experiments were carried out with different concentrations ranging from 0.5–5 mol%. The reactions proceeded faster (approx. 70 s at 5 mol%, 6.6 min at 2.5 mol%, 7.9 min at 2 mol%) with larger catalyst loading. At lower concentrations, however, a minimum concentration of catalyst (>2%) is required to induce complete regeneration of the norbornadiene **1d** (Figure 3). This effect is likely caused by the photobleaching of the photocatalyst.^[^ ^34^ ^]^ Based on the observation that the acridinium ion **4** is able to catalyze the cycloreversion reaction of the quadricyclane **2d**, it was also examined whether structurally resembling annelated quinolizinium ions may also be employed for the cycloreversion reaction.^[^ ^35^ ^]^ Thus, a small series of derivatives **5a**–**5f** ^[^ ^36^ ^]^ was tested as photocatalysts. In general, the cycloreversion of the quadricyclane **2d** was induced upon irradiation in the presence of compounds **5a**–**5f**, as shown by photometric monitoring of the photoreaction (cf. Supporting Information, Figure S5). However, the reactions were very slow under these conditions, and only the 9‐nitrobenzo[*b*]quinolizinium (**5f**) turned out to be an effective catalyst for the cycloreversion (reaction time: ca. 70 s). But even in this case, slightly higher concentrations of the catalyst (7.5 mol%) were required as compared with reactions of the catalyst **4**. Further investigation of the photocatalyzed cycloreversion of **2d** with catalysts **3**, **4**, and **5f** (1 mol%) with in‐situ ^1^H NMR spectroscopy showed the quantitative conversion to the norbornadiene **1d** as well as a slow, but steady decrease of catalyst signals during irradiation (cf. Supporting Information Figures S26–S28).

For comparison, the photocycloreversion of the parent naphthylquadricyclane **2c** to the norbornadiene **1c** was also investigated (Figure 3C). For this purpose, solutions of **2c** and **2d** in the presence of the photocatalyst **4** (5 mol%) were irradiated *(λ*_ex_ = 420 nm) under identical conditions. These results showed that the cycloreversion of the donor‐substituted derivative **2d** (approx. 70 s) is faster than the cycloreversion of **2c** (approx. 5.8 minutes), which supports the proposed initial PET process as essential reaction step (Figure 3D).

The results described above show that the cycloreversion of appropriately substituted quadricyclanes is readily initiated by a suitable photocatalyst in substoichiometric concentrations and with visible light. Although this type of catalyzed cycloreversion is known already for quadricyclanes, the latter cases operate under different conditions. Namely, the photocatalyst unit was either covalently linked to the quadricyclane,^[^ ^27^, ^28^, ^29^ ^]^ or encapsulated in a host system.^[^ ^23a^ ^]^ Or a much larger amount of catalyst (0.5–1 molar equivalent) is required for the cycloreversion reaction.^[^ ^37^ ^]^ In contrast, the system presented herein requires smaller fractions of photocatalysts to initiate the cycloreversion reaction, and the photoreaction proceeds quantitatively. Nevertheless, as a pending drawback, this system cannot be used for a continuous switching between quadricyclane and norbornadiene in its present form because the two applied catalysts for back‐ and forth‐reaction interfere with each other and, more importantly, the quadricyclane can only be stored under sunlight in the absence of the photocatalysts. However, it is conceivable for future applications that the corresponding photocatalysts are immobilized on surfaces^[^ ^38^ ^]^ and kept in separate chambers that are connected through a flow system,^[^ ^7^ ^]^ thus enabling local control of the photoreaction.

**Scheme 4:**
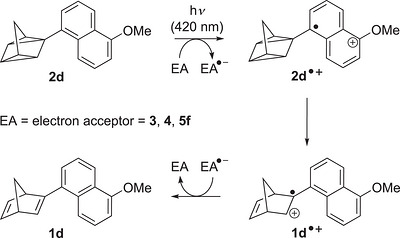
Proposed mechanism of the photocatalyzed cycloreversion of the quadricyclane **2d** to the norbornadiene **1d**.

All observations on the photocatalyzed cycloreversion of the arylquadricyclane **2d** point to a mechanism that is initiated by a PET reaction. Firstly, the catalysts have sufficiently high excited‐state reduction potentials, namely E^*^_red_ = 2.2 V (**3**) (versus NHE),^[^ ^39^ ^]^ E^*^_red_ = 2.4 V (versus NHE) (**4**),^[^ ^39^ ^]^ and E^*^_red_ = 2.7 (versus NHE) (**5f**) (cf. Supporting Information, Figure S29). At the same time, the oxidation potential of the naphthylquadricyclane **2d** is estimated to be in the range of the one of 1‐methoxynaphthalene (*E*_ox_ = 1.6 V versus NHE^[^ ^40^ ^]^), with the naphthyl unit as the electron acceptor. Based on these data, the PET reaction between the photocatalysts and the naphthylquadricyclane is thermodynamically favored, which is in agreement with established PET processes and radical ion formation of these components.^[^ ^41^ ^]^ This interpretation is also in line with the observation that the photocatalyzed cycloreversion of the quadricyclane **2c** is significantly slower than the one of **2d**, because the naphthalene unit without the methoxy group is supposed to have a higher oxidation potential, likely similar to the one of naphthalene (*E*_ox_ = 1.9 V versus NHE^[^ ^42^ ^]^). According to the proposed mechanism, as deduced from the already established formation and reactivity of radical cations,^[^ ^20^, ^23^, ^24^, ^25^, ^26^, ^27^, ^28^, ^29^ ^]^ the photoinduced electron transfer between the excited photocatalyst and the quadricyclane **2d** leads to the formation of a radical cation **2d** ^•+^ and the radical anion of the electron acceptor **EA** ^•–^ (Scheme 4, note that the acridinium and benzoquinolizinium units result in neutral radicals). As the norbornadiene **1d** is formed without any byproducts resulting from other radical ion intermediates, a concerted (but still asynchronous) cycloreversion to the radical cation of the norbornadiene, **1d** ^•+^ is assumed, thus resembling the kinetically favorable reaction pathway of the parent compound.^[^ ^20^ ^]^ Finally, electron back transfer from the reduced catalyst **EA^•−^** takes place and the norbornadiene **1d** is formed along with regeneration of the catalyst (Scheme 4). The observation, that the benzoquinolizinium derivatives **5a**–**e** do not operate as efficient photocatalysts, even though they are strong electron acceptors in the excited state, may be explained by additional, highly competing deactivation pathways of the excited state. In fact, it has been shown already for these derivatives that emission, fast intersystem crossing and even photodimerization take place upon irradiation,^[^ ^43^ ^]^ which strongly interferes with the photocatalytic activity.

As a control experiment to support the proposed mechanism, the cycloreversion was also initiated independently by the reaction of **2d** with the known ground‐state electron acceptor magic blue^[^ ^19^, ^20^ ^]^ in MeCN‐*d*_3_, which led also to a quantitative cycloreversion of **2d** to the norbornadiene **1d**, as shown by ^1^H NMR‐spectroscopic analysis of the reaction mixture (cf. Supporting Information, Figure S23). As it is known that this ground‐state reaction between quadricyclane derivatives and magic blue or resembling triarylammonium ions initially leads to the corresponding radical cation of the quadricyclane by an electron transfer reaction,^[^ ^20^ ^]^ this result provides further evidence that the PET induced cycloreversion proceeds through the formation of the same intermediates.

Although the PET‐induced cycloreversion is only shown with two particular examples herein, it is proposed that this reaction may be applied as a general route for the efficient and fast transformation of aryl‐substituted quadricyclanes to the corresponding norbornadienes, as long as the redox potentials of the aryl substituent, i.e., the electron donor, and the applied photocatalyst match (see above). Even additional substitution at the norbornadiene scaffold may be tolerated, which is an important feature considering the fact that several promising norbornadienes for MOST applications carry cyano groups at the alkene unit.^[^ ^12g^ ^]^ For example, the reported oxidation potentials of 3‐aryl‐2‐cyanoquadricyclanes^[^ ^15^ ^]^ or resembling derivatives^[^ ^16c,d^ ^]^ indicate that the triggering PET reaction with a photocatalyst should be thermodynamically favorable. In addition, it has been demonstrated also that the electrochemical formation of the corresponding radical cation induces the cycloreversion reaction as well.

## 3 Conclusion

In summary, we showed in a case study that donor‐substituted arylquadricyclanes may operate as an ideal substrate for a PET‐triggered cycloreversion to the corresponding norbornadienes. Specifically, we identified suitable PET catalysts that initiate the fast cycloreversion of the quadricyclane **2d** to the norbornadiene **1d** with visible light (420 nm) and with relatively low catalyst loading. At the same time, there is no significant loss of favorable MOST properties, that is, half‐life and energy storage density. Most notably, the option to initiate the cycloreversion of the quadricyclane with visible light in addition to its already established photoinduced formation with visible light underlines the utility and versatility of this class of norbornadiene‐quadricyclane systems for MOST applications. In particular, the presented approach may provide a convenient tool for the targeted release of stored energy from the quadricyclane with high temporal and local control.

## Supporting Information

The authors have cited additional references within the Supporting Information.^[^ ^44^, ^45^, ^46^, ^47^, ^48^ ^]^

## Author Contributions

Author names are in alphabetical order that does not reflect the specific contribution of each author.

## Conflict of Interest

The authors declare no conflict of interest.

## Supporting information



Supporting Information

## Data Availability

Data sharing is not applicable to this article as no new data were created or analyzed in this study.
