# Supplementary material for: Photoinduced Electron Transfer (PET) as Key to Accelerate the Cycloreversion Reaction of Arylquadricyclanes
Source: Chemistry. 2025 Sep 29;32(28):e02413. doi: 10.1002/chem.202502413 (PMC13411375; doi:10.1002/chem.202502413)
Supplement: Supplementary file 1 — Supporting Information [file CHEM-32-e02413-s001.pdf]

Supporting Information

Table of Contents

1. Equipment S3
2. Methods S3
3. Materials S3
4. Synthesis S4
5. Absorption properties S7
6. Photoisomerization quantum yield S8
7. Photoreactions S9
8. Reaction of quadricyclane 2d with magic blue S12
9. Kinetic studies of the thermally induced cycloreversion S12
10. DSC measurements S13
11. NMR spectra S14
12. In-situ NMR spectroscopy S23
13. Cyclovoltammetric analysis S26
14. References S27

1. Equipment

The NMR spectra were recorded on a JEOL ECZ 500 spectrometer (^{1}H: 500 MHz, ^{13}C: 125 MHz, 25 °C) or on a Varian VNMR-S 600 (^{1}H: 600 MHz, 13C: 150 MHz, 25 °C). The spectra were referenced to the residual signals of MeCN-d_3 [δ (^{1}H) = 1.94 ppm, δ(^{13}C) = 1.32 ppm], $CDCl_3$ [δ(^{13}C) = 77.16 ppm], C_6D_6 [δ(^{1}H) = 7.16 ppm, δ(^{13}C) = 128.1 ppm], or the internal standard tetramethylsilane (TMS) [δ(^{1}H) = 0.00 ppm] included in the $CDCl_3$, and processed with the MestReNova software. The melting points were determined with BÜCHI 545 (Büchi, Flawil, CH) and are uncorrected. Elemental analysis data were determined in-house (Organic Chemistry, University of Siegen) on a HEKAtech EUROEA combustion analyzer. The absorption spectra were measured on a Varian Cary 100 Bio absorption spectrometer or on an Analytik Jena SPECORD S spectrometer with Hellma quartz glass cuvettes 115 F-QS (d = 10 mm). The absorption spectra were processed with the software Origin (OriginPro 8.5.1) with the implemented smoothing function "adjacent averaging" with the factor of 10. The fluorescence spectrum was recorded on Varian Cary eclipse with a Hellma quartz glass cuvette 111-QS (d = 10 mm). Photoreactions were performed with Thorlabs LED M310L1 [310 nm, (56.5 mW)], Thorlabs LED M340L5 [340 nm, (69.2 mW)] or a LUMOS 43 [275 nm (10 mW), 315 nm (10 mW), 360 nm (200 mW/cm^2), 420 nm (200 mW/cm^2)] from Atlas Photonics and self-build 520 nm LED (Conrad electronic Nr. 181862, 3.8 V, 1000 mA). In-situ NMR irradiations were performed with 405 nm and 520 nm LEDs.[32] Differential scanning calorimetry was performed on a DSC 204 (Netzsch), with aluminium pans with pierced lids in continuous N_2 flow. The cyclovoltammetric measurement was performed with a PARASTAT® 2273 (Princeton Applied Research) in anhydrous DMF with a Pt-disk (⌀ = 1 mm) as the working electrode, silver wire as the pseudo reference electrode, platinum wire as the counter electrode and with tetrabutylammonium hexafluorophosphate (0.1 M) as the supporting electrolyte. The sample was measured against ferrocene ($E_{1/2}$ = +0.32 V) as the internal standard with a scan rate of 0.1 V/s.

2. Methods

Reaction mixtures were stirred with a magnetic stirring bar (400–750 rpm). Solvents were removed with a rotary evaporator at 20–40 °C under reduced pressure (360–15 mbar). Air-sensitive reactions were performed under an inert atmosphere (Ar) with Schlenk equipment. Solvents/solutions were deaerated by passing Argon trough the solution (approx. 5 min) prior their use. Room temperature (r.t.) was between 20 °C and 25 °C.

3. Materials

Commercially available chemicals were purchased from AnalytiChem GmbH (K_2CO_3), BLD Pharmtech Ltd.(5-bromonaphthalen-1-amine), Carbolution Chemicals GmbH [$Pd(PPh_3)_4$], Th. Geyer GmbH & Co KG (glacial acetic acid, $NaNO_2$), Fisher Scientific GmbH [MeI, HBF_4 (50 w/w%), 9,10-dicyanoanthracene], Merck KGaA (9-mesityl-10-methylacridinium perchlorate). 4,4,5,5-Tetramethyl-2-(bicyclo[2.2.1]heptadien-2-yl)-1,3,2-dioxaborolane (**1b**),[30b] tetracyclo-[3.2.0.0^{2,7}.0^{4,6}]heptylnaphthalene (**2c**),[11, 30b] 5-bromo-1-hydroxynaphthalene,[44] benzo[*b*]quinolizinium tetrafluoroborate (**5a**),[36a] 9-fluoro-benzo[*b*]quinolizinium bromide, [36b] 9-chlorobenzo[*b*]quinolizinium bromide,[36b] 9-bromobenzo[*b*]quinolizinium bromide,[36b] 9-methoxybenzo[*b*]quinolizinium bromide,[36d] and 9-nitrobenzo[*b*]quinolizinium perchlorate (**5f**) [36c] were synthesized according to literature.

n-Hexane was purified by distillation prior to use. The DMF for the cyclovoltammetric measurements was dried with CaH_2 and purified by subsequent fractional distillation under reduced pressure. All other reactants and solvents were used without purification. The silica used for column chromatography was purchased from Machery-Nagel GmbH & Co KG (particle size 250 µm).

4. Synthesis

1-Bromo-5-methoxynaphthalene

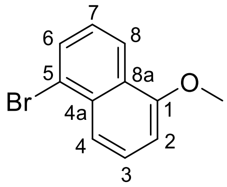

A suspension of 5-bromo-1-hydroxynaphthalene (1.00 g, 4.48 mmol), MeI (0.6 mL, 1.4 g, 10 mmol) and K_2CO_3 (772 mg, 5.59 mmol) in DMF (8.0 mL) was stirred at r.t. for 3 d. Then, water (40 mL) was added and the resulting suspension was stirred until the initial formed droplets solidified. The resulting precipitate was filtered off and dried under reduced pressure. The product was obtained as a beige microcrystalline powder (1.01 g, 4.26 mmol, 95%), mp 66–68 °C (lit.: oil[44]). – ^{1}H NMR (500 MHz, $CDCl_3$): δ = 4.00 (s, 3H, CH_3), 6.87 (dd, ^3J = 7.7 Hz, ^4J = 0.9 Hz, 1H, 2-H), 7.29 (dd, ^3J = 8.5 Hz, ^3J = 7.4 Hz, 1H, 7-H), 7.48 (dd, ^3J = 8.6 Hz, ^3J = 7.7 Hz, 1H, 3-H), 7.78 (dd, ^3J = 7.4 Hz, ^4J = 1.2 Hz, 1H, 6-H), 7.81 (dt, ^3J = 8.6 Hz, ^4J = 0.9 Hz, 1H, 4-H), 8.26 (dt, ^3J = 8.4 Hz, ^4J = 1.0 Hz, 1H, 8-H). – ^{13}C-NMR (125 MHz, $CDCl_3$): δ = 55.9 (OCH_3), 104.7 (C2), 119.4 (C4), 122.1 (C8), 122.6 (C5), 125.5 (C7), 127.0 (C8a), 127.4 (C3), 130.7 (C6), 133.2 (C4a), 155.6 (C1).

*1-(Bicyclo[2.2.1]hepta-2,5-dien-2-yl)-5-methoxynaphthalene (**1d**)*

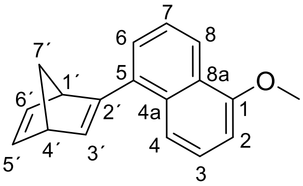

An mixture of 1-bromo-5-methoxynaphthalene (191 mg, 806 µmol), 4,4,5,5-tetramethyl-2-(bicyclo[2.2.1]-heptadien-2-yl)-1,3,2-dioxaborolane (**1b**) (193 mg, 886 µmol), $Pd(PPh_3)_4$ (46.8 mg, 40.5 µmol, 5 mol%), THF (6.4 mL), and aq. NaOH (8.4 mmol, 2.7 M, 3.1 mL) was stirred at 80° for 16 h under anaerobic conditions. After cooling the emulsion to room temperature, EtOAc (15 mL) was added and the organic layer was separated and dried with Na_2SO_4. The drying agent was filtered off, the solvent was removed under reduced pressure, and the crude product was purified with column chromatography (SiO_2, *n*-hexane/EtOAc, 99/1, *v*/*v*, R_f = 0.56). The product was obtained as a colourless viscous liquid which solidified slowly to a white amorphous solid (166 mg, 668 µmol, 83%), mp 69–70 °C. – ^{1}H NMR (500 MHz, $CDCl_3$): δ = 2.16 (dt, ^2J = 6.0 Hz, ^3J = 1.5 Hz, 1H, 7´-H), 2.37 (dt, ^2J = 6.0 Hz, ^3J = 1.5 Hz, 1H, 7´-H), 3.79–3.83 (m, 2H, 1´-H, 4´-H), 4.00 (s, 3H, OCH_3), 6.79 (d, ^3J = 3.1 Hz, 1H, 3´-H), 6.82 (dd, ^3J = 7.7 Hz, ^3J = 0.9 Hz 1H, 2-H), 6.87 (dd, ^3J = 5.2 Hz, ^3J = 3.0 Hz, 1H, 5´-H), 7.05 (dd, ^3J = 5.2 Hz, ^3J = 3.0 Hz, 1H, 6´-H), 7.27 (dd, ^3J = 7.0 Hz, ^4J = 1.1 Hz, 1H, 6-H), 7.35 (dd, ^3J = 8.5 Hz, ^3J = 7.7 Hz, 1H, 3-H), 7.40 (dd, ^3J = 8.4 Hz, ^3J = 7.0 Hz, 1H, 7-H), 7.68 (dd, ^3J = 8.6 Hz, ^4J = 0.9 Hz, 1H, 4-H), 8.17 (dd, ^3J = 8.4 Hz, ^4J = 1.1 Hz, 1H, 8-H). – ^{13}C-NMR (500 MHz, $CDCl_3$): δ = 51.5 (C1´), 55.7 (OCH_3), 56.2 (C4´), 73.5 (C7´), 103.9 (C2), 118.3 (C4), 121.2 (C8), 124.0 (C6), 124.8 (C7), 125.8 (C3), 126.2 (C8a), 132.3 (C4a), 136.0 (C5), 139.9 (C3´), 142.9 (C6´), 143.4 (C5´), 155.8 (C1), 156.5 (C2´). – El. Anal. for $C_{18}H_{16}O$: calc. (%): C 87.06, H 6.49; found (%): C 87.23, H 6.62.

*Tetracyclo[3.2.0.0^{2,7}.0^{4,6}]heptyl-5-methoxynaphthalene (**2d**)*

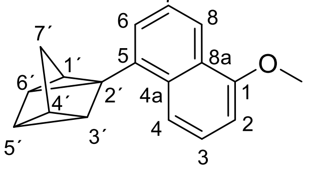

A stirred solution of **1d** (50.0 mg, 201 µmol), Ru(phen)$_3$(PF$_6$)$_2$ (1.88 mg, 2.01 µmol, 1 mol%) in deaerated MeCN (5 mL) was irradiated with λ_{ex} = 520 nm at r.t. for 90 min. The solvent was removed under reduced pressure at 20 °C. The residue was suspended in *n*-pentane (10 mL) and filtered. The solvent was removed under reduced pressure, and the product was obtained as a colourless oil, which solidified over days to an amorphous solid (47.3 mg, 190 µmol, 95%). – ^{1}H NMR (500 MHz, CDCl$_3$): δ = 1.65 (dt, ^2J = 4.9 Hz, ^3J = 1.4 Hz, 1H, 1´-H), 1.70–1.75 (m, 1H, 4´-H), 1.78–1.83 (m, 1H, 5´-H), 1.90–1.95 (m, 1H, 3´-H), 2.19–2.26 (m, 2H, 7´-H, 6´-H), 2.43–2.47 (m, 1H, 7´-H), 3.99 (s, 3H, OCH$_3$), 6.82 (d, ^3J = 7.6 Hz, 1H, 2-H), 7.26 (d, ^3J = 7.0 Hz, ^4J = 1.2 Hz, 1H, 6-H), 7.34 (dd, ^3J = 8.4 Hz, ^3J = 7.0 Hz, 1H, 7-H), 7.40 (dd, ^3J = 8.5 Hz, ^3J = 7.6 Hz, 1H, 3-H), 7.66 (dt, ^3J = 8.5 Hz, ^4J = 0.9 Hz, 1H, 4-H), 8.13 (dt, ^3J = 8.4 Hz, ^4J = 1.2 Hz Hz, 1H, 8-H). – ^{13}C NMR (125 MHz, CDCl$_3$) δ = 13.9 (C5´), 21.0 (C6´), 21.7 (C3´), 25.4 (C4´), 30.9 (C2´), 32.2 (C1´), 32.9 (C7´), 55.7 (OCH$_3$), 103.9 (C2), 118.2 (C4), 120.8 (C8), 124.9 (C7), 125.7 (C3), 125.9 (C6), 126.0 (C8a), 134.5 (C4a), 136.2 (C5), 156.0 (C1).

*General procedure (GP A) for the precipitation of benzo[b]quinolizinium tetrafluoroborates **5b**–**5e***

An aqueous solution of HBF$_4$ (50 w/w%) was added to an aqueous solution of the benzo[*b*]quinolizinium bromide. The resulting precipitate was filtered off and dried under reduced pressure. ^{1}H NMR spectra of the tetrafluoroborates **5b**–**5d** were in accordance with the ones of the bromide salts.[36b]

*9-Fluorobenzo[b]quinolizinium tetrafluoroborate (**5b**)*

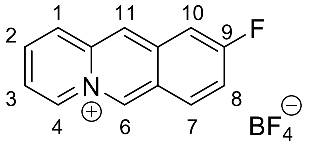

5b

The product was synthesized from 9-fluorobenzo[*b*]quinolizinium bromide according to GP A. The product was obtained as an amorphous yellow powder, mp 176–177 °C. – ^{1}H NMR (500 MHz, DMSO-*d*$_6$) δ = 7.92–7.98 (m, 1H, 3-H), 7.95–8.02 (m, 1H, 8-H), 8.10 (ddd, ^3J = 7.9 Hz, ^3J = 6.9 Hz, ^4J = 0.9 Hz, 1H, 2-H), 8.24 (dd, ^3J = 9.5 Hz, ^4J = 2.5 Hz, 1H, 10-H), 8.57 (d, ^3J = 8.9 Hz, 1H, 1-H), 8.64 (dd, ^3J = 9.5 Hz, ^3J = 5.5 Hz, 1H, 7-H), 9.16 (s, 1H, 11-H), 9.27 (d, ^3J = 7.0 Hz, 1H, 4-H), 10.47 (s, 1H, 6-H, 6-H). – EI. Anal. for $C_{13}H_9BF_5N$: calc. (%): C 54.78, H 3.18, N 4.91; found (%): C 54.56, H 3.04, N 4.70.

*9-Chlorbenzo[b]quinolizinium tetrafluoroborate (**5c**)*

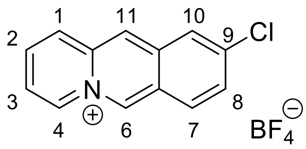

5c

The product was synthesized from 9-chlorobenzo[*b*]quinolizinium bromide according to GP A. The product was obtained as an amorphous yellow powder, mp 212–213 °C. – ^{1}H NMR (500 MHz, DMSO-d_6) δ = 7.97–8.00 (m, 1H, 3-H), 8.01 (dd, ^3J = 9.3 Hz, ^4J = 1.8 Hz, 2H, 8-H), 8.12 (ddd, ^3J = 8.9 Hz, ^3J = 6.9 Hz, ^4J = 1.1 Hz, 1H, 2-H), 8.53 (d, ^3J = 9.3 Hz, 1H, 7-H), 8.58 (d, ^4J = 1.8 Hz, 1H, 10-H), 8.60 (dd, ^3J = 9.0 Hz, ^4J = 0.9 Hz, 1H, 1-H), 9.14 (s, 1H, 11-H), 9.28 (d, ^3J = 6.7 Hz, 1H, 4-H), 10.45 (s, 1H, 6-H). – El. Anal. for $C_{13}H_9BClF_4N$: calc. (%): C 51.79, H 3.01, N 4.65; found (%): C 51.99, H 2.82, N 4.67.

*9-Brombenzo[b]quinolizionium tetrafluoroborate (**5d**)*

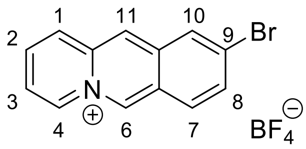

5d

The product was synthesized from 9-bromobenzo[*b*]quinolizinium bromide according to GP A. The product was obtained as an amorphous yellow powder, mp 210–212 °C. – ^{1}H NMR (500 MHz, DMSO-d_6) δ = 7.99 (td, ^3J = 7.0 Hz, ^4J = 1.5 Hz, 1H, 3-H), 8.08–8.15 (m, 2H, 2-H, 8-H), 8.43 (d, ^3J = 9.2 Hz, 1H, 7-H), 8.60 (d, ^3J = 8.8 Hz, 1H, 1-H), 8.75 (d, ^4J = 1.1 Hz, 1H, 10-H), 9.13 (s, 1H, 11-H), 9.27 (d, ^3J = 6.8 Hz, 1H, 4-H), 10.43 (s, 1H, 6-H). – El. Anal. for $C_{13}H_9BBrF_4N$: calc. (%): C 45.14, H 2.62, N 4.05; found (%): C 45.16, H 2.53, N 3.81.

*9-Methoxybenzo[b]quinolizionium tetrafluoroborate (**5e**)*

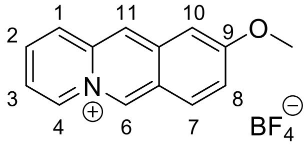

5e

The product was synthesized from 9-Methoxybenzo[*b*]quinolizinium bromide according to GP A. The product was obtained as an amorphous yellow powder, mp 169–173 °C. – ^{1}H NMR (500 MHz, DMSO-d_6) δ = 4.08 (s, 3H, OCH_3), 7.67 (dd, ^3J = 9.2, ^4J = 2.4 Hz, 1H, 8-H), 7.70 (d, ^4J = 2.4 Hz, 1H, 10-H), 7.80 (td, ^3J = 6.9 Hz, ^4J = 1.4 Hz, 1H, 3-H), 7.98 (m, 1H, 2-H), 8.41 (d, ^3J = 9.2 Hz, 1H, 7-H), 8.44 (d, ^3J = 9.0 Hz, 1H), 8.90 (s, 1H, 11-H), 9.12 (d, ^3J = 6.9 Hz, 1H, 4-H), 10.23 (s, 1H, 6-H). – El. Anal. for $C_{14}H_{12}BF_4NO$: calc. (%): C 56.61, H 4.07, N 4.72; found (%): C 56.16, H 3.78, N 4.46.

5. Absorption properties

Solutions (c = 20 µM, V = 2.00 mL) of the norbornadiene **1d** in MeCN, EtOH, $CHCl_3$, EtOAc, benzene and cyclohexane were prepared from a stock solution of **1d** in cyclohexane (c = 1 mM), and the absorption spectrum was subsequently measured (Figure 1).

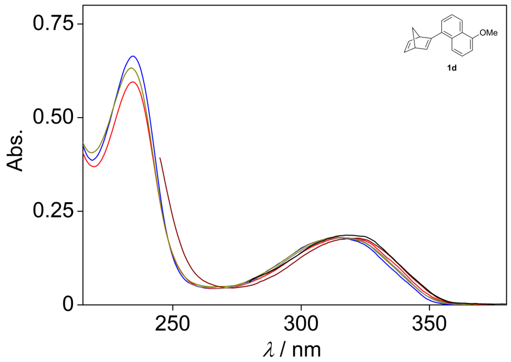

Figure S1. Absorption spectra of **1d** (c = 20 µM) in MeCN (—), EtOH (—), $CHCl_3$ (—), EtOAc (—), benzene (—) and cyclohexane (—).

Solutions (c = 20 µM, V = 2.00 mL) of the photocatalysts Ru(phen)$(PF_6)_2$, **3**, **4** and **5a–f** in MeCN were prepared from their stock solutions in MeCN (c = 1.00 mM) except for **3**, which was prepared form a stock solution in $CHCl_3$ (c = 1.00 mM). The absorption spectrum was subsequently measured (Figure 2).

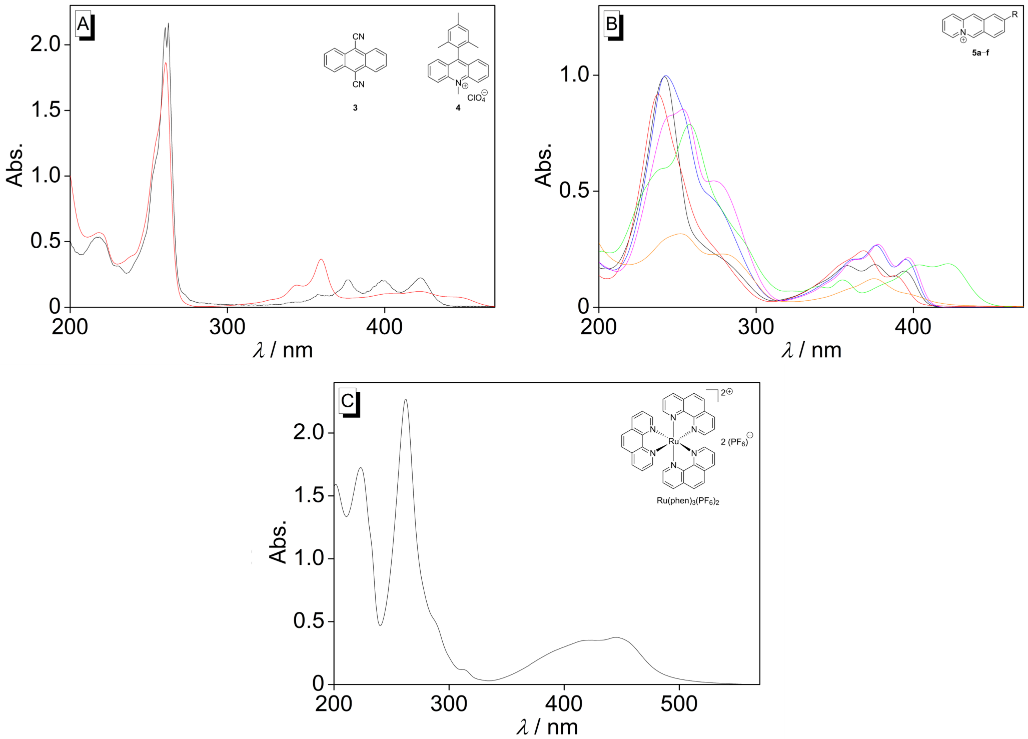

Figure S2. Absorption spectra of PET photocatalysts **3** (—), **4** (—) (A); **5a** (—), **5b** (—), **5c** (—), **5d** (—), **5e** (—), and **5f** (—) (B); and Ru(phen)$(PF_6)_2$ (—) (C) in MeCN (c = 20 µM).

6. Photoisomerization quantum yield

The quantum yield of the cycloaddition was determined by the irradiation of potassium ferri-oxalate $K_3[Fe(C_2O_4)_3]$ in the presence of 1,10-phenanthroline with Thorlabs M310L1 LED (λ_{ex} = 310 nm).[45] The photon flux of the LED was determined according to literature and revealed a light intensity of $I_0 = 8.53 \times 10^{-8}$ einstein L^{-1} s^{-1} (Figure 3 A).[30d, 45] The photo-isomerization quantum yield was determined by literature procedure (Regime 1: Total absorption regime).[46] The absorbance of the prepared solutions was adjusted as Abs. >2 at the excitation wavelength to ensure a linear dependence between the decreasing absorption and irradiation time (Figure 3 B).[46]

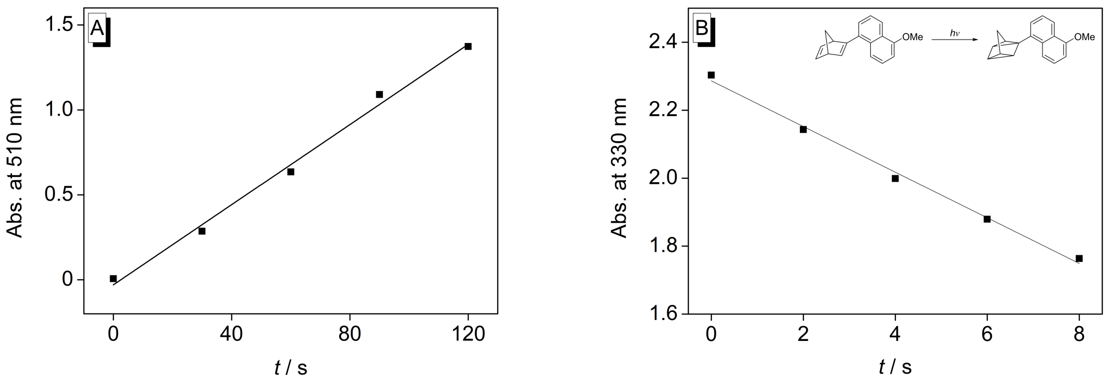

Figure S3. Determination of photon flux for Thorlabs M310L1 LED (A) taken from Ref [30d] and photometric monitoring of the photoreaction of **1d** (B). The straight lines represent the linear fit of the experimental data.

7. Photoreactions

Photometric investigation of the cycloaddition and cycloreversion reaction

The cycloaddition was investigated photometrically by the direct irradiation of a solution of the norbornadiene **1d** (c = 20 µM, V = 2.00 mL, MeCN) either with λ_{ex} = 315 nm light or with λ_{ex} = 520 nm light in the presence of $Ru(phen)_3(PF_6)_2$ (1 equiv., c = 20 µM). The irradiation was stopped, when no further change in absorption was detectable.

The cycloreversion was also investigated by irradiation of a solution of **2c** or **2d** (c = 20 µM, V = 2.00 mL) and photocatalyst **3**, **4**, or **5a**–**f** (0.5%–10%) with λ_{ex} = 420 nm. The reaction progress was investigated photometrically. The irradiation was stopped, when no further change in absorption occurred. In every photometric investigation, the absorption of the photocatalysts **3**, **4**, or **5a**–**f** were subtracted, leaving only the absorption of the quadricyclanes **2c** or **2d** visualized. The cycloreversion of **2d** was studied with different concentrations (0.5–5 %) of the catalyst **4** (Figure 4).

^{1}H NMR-spectroscopic investigation of the cycloaddition and cycloreversion reaction

Solutions of **1d** (c = 9 mM) were irradiated with LUMOS 43 at λ_{ex} = 315 nm (Figure 23). The photoisomerization with $Ru(phen)_3(PF_6)_2$ was followed by in-situ NMR spectroscopy[32] at λ_{ex} = 520 nm (60.5 mW optical energy) (Figure 25).

*General procedure (GP B) for the preparation of quadricyclane **2d** solutions for ^{1}H NMR spectroscopy*

A solution of the norbornadiene **1d** (c = 60 mM, V = 600 µL, MeCN-d_3) was irradiated with λ_{ex} = 340 nm for 16–20 min. The solution contained 95 ± 3% of quadricyclane **2d**.

The cycloreversion reaction of **2d** (c = 60 mM, V = 600 µL, MeCN-d_3) in the presence of **3**, **4** or **5f** (1 mol%) was investigated with in-situ ^{1}H NMR spectroscopy and irradiation with λ_{ex} = 405 nm (2.1 mW optical energy) (Figure 26–28).

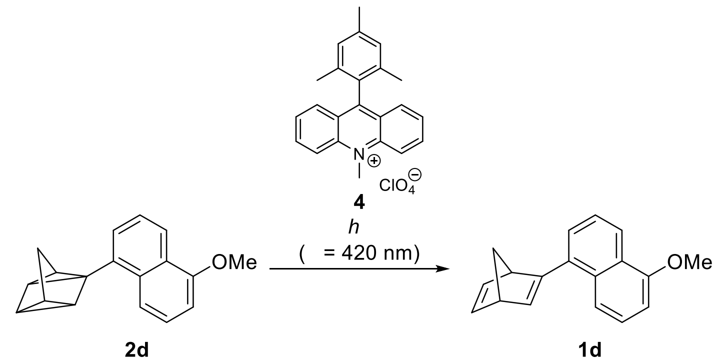

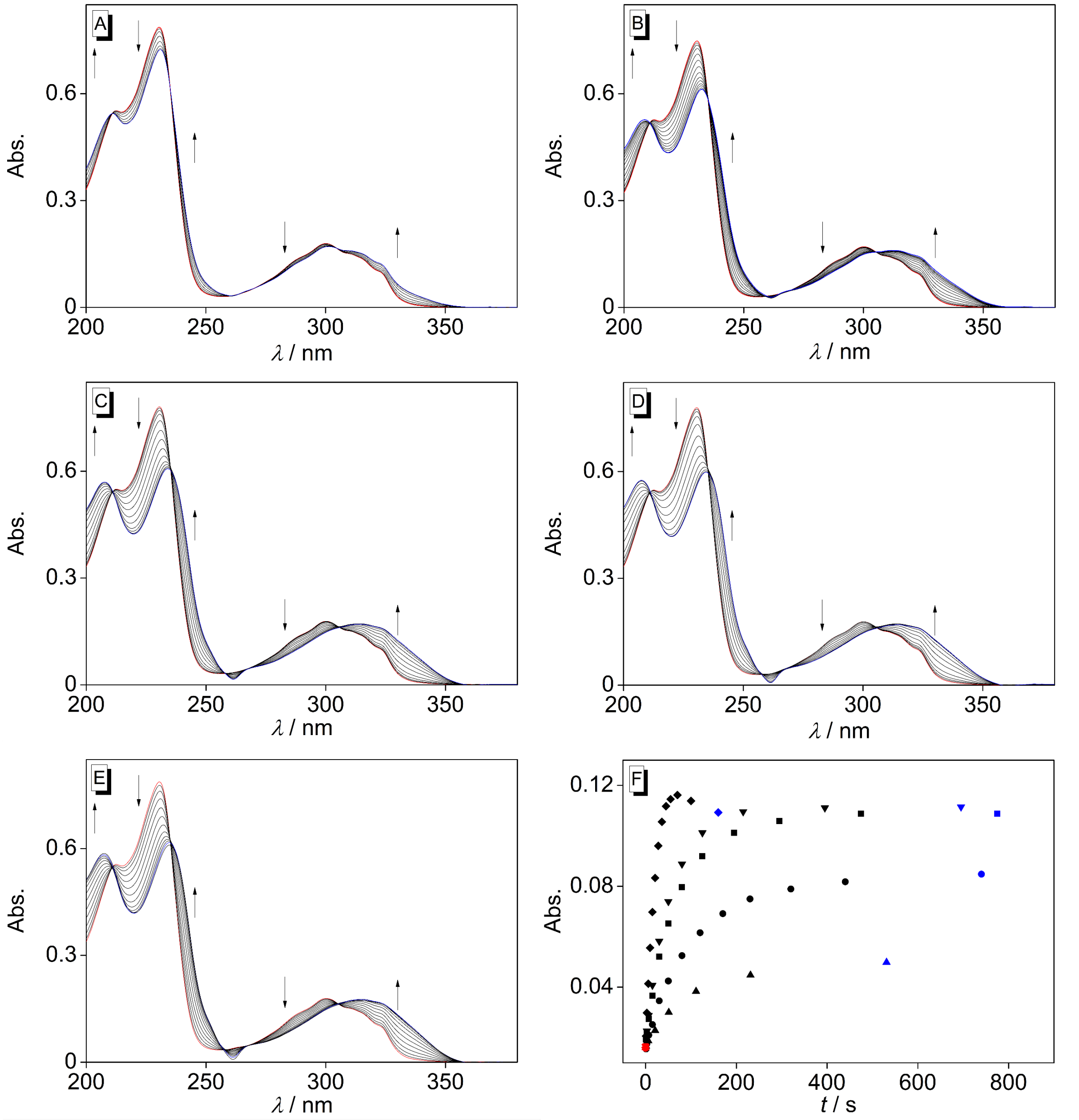

Figure S4. Photometric monitoring of the photoinduced cycloreversion of **2d** (c = 20 µM) with the photocatalyst **4** with 0.5% (A), 1% (B), 2% (C), 2.5% (D) and 5% (E) in MeCN at λ_{ex} = 420 nm. Plot of absorption at 333 nm versus reaction time (F) for the cycloreversion of **2d** at λ_{ex} = 420 nm with varying concentration of **4** (0.5% = ▲, 1% = ●, 2% = ■, 2.5% = ▼, 5 % = ♦). Red represents the quadricyclane **2d**, blue indicates the endpoint of conversion to the norbornadiene **1d**.

Catalyst screening

The benzo[*b*]quinolizinium derivatives **5a–f** were investigated with regards to their propensity as photocatalyst for the cycloreversion reaction (Figure 6). A solution of **1d** (V = 1.96 mL, $c \approx$ 20 μM) was irradiated with λ_{ex} = 315 nm until the PSS was reached (3 min). Then the photocatalyst was added (V = 4 μL, c = 1 mM). The obtained solution (V = 2.00 mL, c_{2d} = 20 μM, c_{5a-f} = 2 μM) was irradiated at λ_{ex} = 420 nm until no further change in absorption occurred was detected.

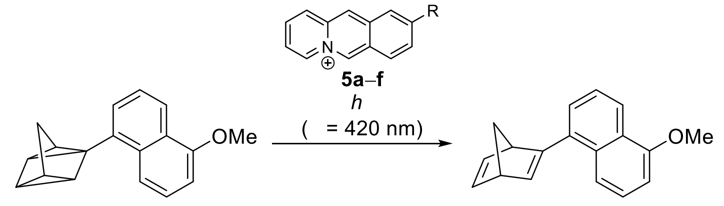

2d **1d**

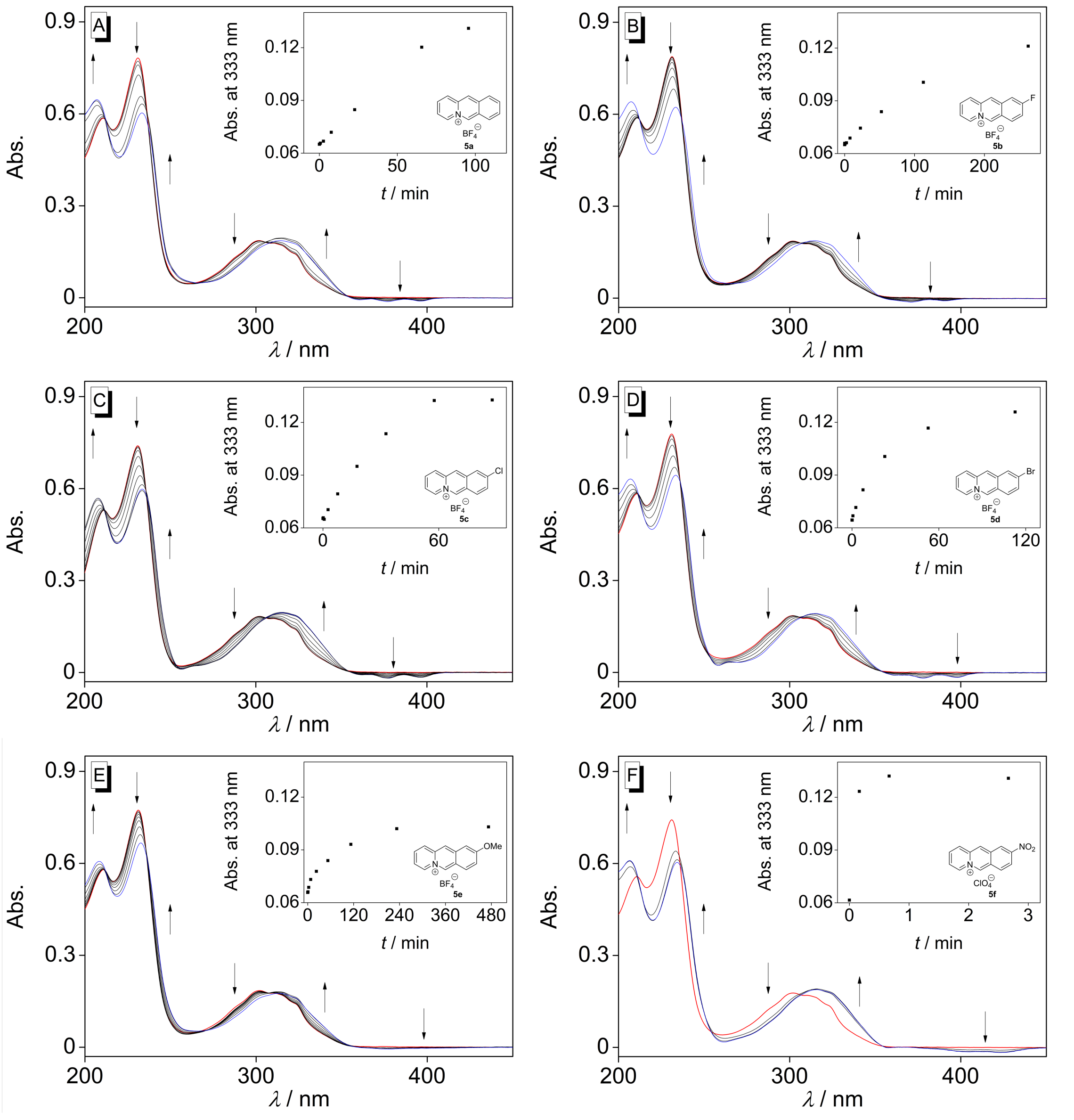

Figure S5. Photometric monitoring of the photoinduced cycloreversion of **2d**/**1d** (72/28) (c = 20 μM) with the catalysts **5a**–**f** (A–F) (10 mol%) in MeCN at λ_{ex} = 420 nm. Insets: plot of absorption at 333 (A–F) versus reaction time.

8. Reaction of quadricyclane 2d with magic blue

A quadricyclane solution **2d**, prepared according to GP B, was quickly added to a solution of magic blue in MeCN-d_3 (1 mol%, 0.30 mg in 50 µL). The ^{1}H NMR spectrum showed complete conversion to the norbornadiene **1d** (Figure 24).

9. Kinetic studies of the thermally induced cycloreversion

The thermally induced cycloreversion of **2d** was monitored by absorption spectroscopy (Figure 6). Therefore, a solution of the norbornadiene **1d** (c = 20 µM, toluene) was irradiated for 5 min with λ_{ex} = 340 nm to obtain the corresponding quadricyclane **2d**. The absorption increase ($\lambda_{abs.}$ = 330 nm) of the norbornadiene was determined at 80 °C.

Considering, that the cycloreversion is a monomolecular reaction, first-order reaction kinetics are applied for further analysis (eq. 1).

$$-\frac{\mathrm{d}\,[\mathrm{QC}]}{\mathrm{d}t}=\frac{\mathrm{d}\,[\mathrm{NBD}]}{\mathrm{d}t}=k \qquad \text{(eq. 1)}$$

In equation 1, [QC] is the concentration of the quadricyclane, [NBD] is the concentration of the norbornadiene and t is the time. k describes the rate constant of the cycloreversion at a certain temperature and can be obtained by an exponential fit.

With the previous determined rate constant k and the Eyring-equation, the Gibbs-energy $\Delta G^{\ddagger}$ and the rate constant for T = 25 °C can be determined (eq. 2 and 3).

$$k=\frac{k_\mathrm{B}T}{h}\mathrm{e}^{-\frac{\Delta G^{\ddagger}}{RT}} \qquad \text{(eq. 2)}$$

$$\Delta G^{\ddagger}=RT\left(23.760+\ln\left(\frac{T}{k}\right)\right) \qquad \text{(eq. 3)}$$

In the equations 2 and 3, k_B is the Boltzmann constant, T is the temperature, h is the Planck constant and R is the universal gas constant.

With the temperature adjusted rate constant k, the half-life of the quadricyclane **2d** was determined (eq. 4).

$$t_{1/2}=\frac{\ln(2)}{k} \qquad \text{(eq. 4)}$$

In equation 4, $t_{1/2}$ is the half-life and k is the rate constant of the cycloreversion at T = 25 °C

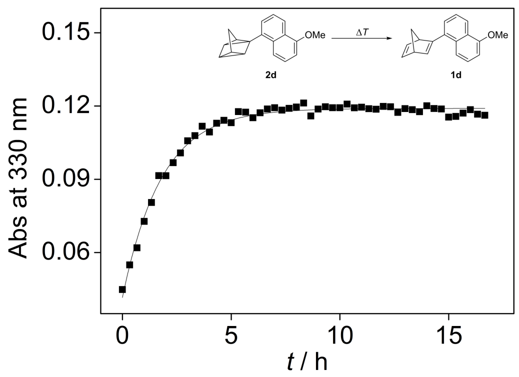

Figure S6. Photometric analysis of the thermally induced cycloreversion of quadricyclane **2d** at T = 80 °C in toluene. The line represents the fit of the experimental data to the exponential function (eq.1).

10. DSC measurements

The quadricyclane **2d** (2.95 mg) was sealed in an aluminum crucible with a pierced lid and put in the calorimeter at room temperature under a nitrogen atmosphere. The following temperature program was used to measure the heat release.[30a]

1. Maintain 25 °C for 15 min
2. Heating from 25 °C to 250 °C with 5.0 °C min^{-1}
3. Cooling to 70 °C with 5.0 °C min^{-1}
4. Heating from 70 °C to 250 °C with 5.0 °C min^{-1}
5. Cooling to 25 °C with 5.0 °C min^{-1}

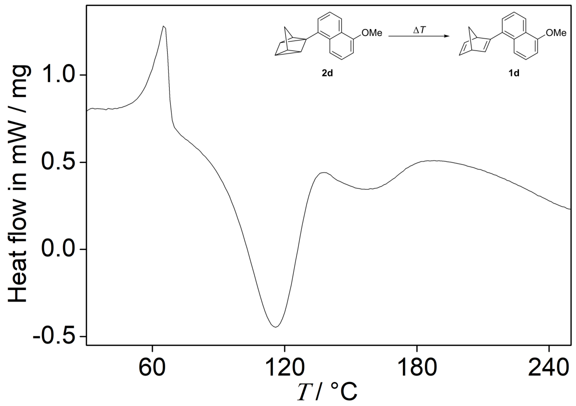

Figure S7. Thermogram from the DSC measurement of the quadricyclane **2d**. Positive peaks represent endothermic processes, while negative signals represent exothermic processes.

11. NMR spectra

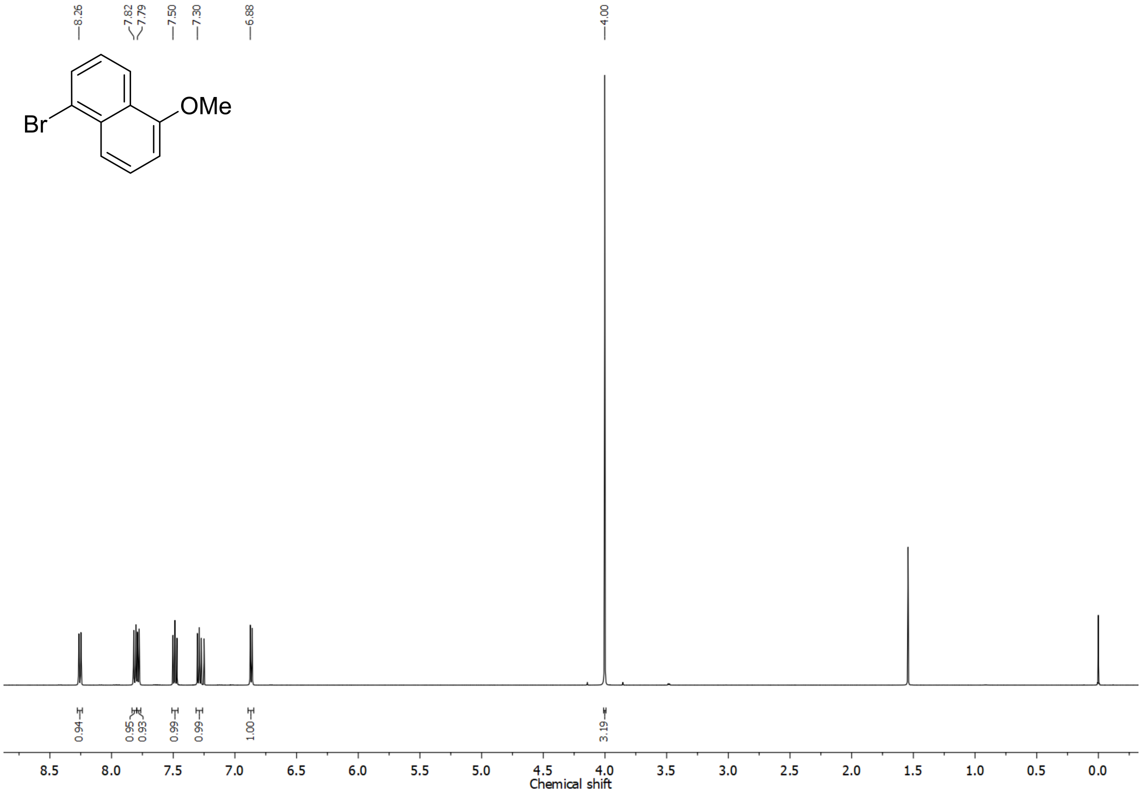

Figure S8. ^{1}H NMR spectrum (500 MHz) of 1-bromo-5-methoxynaphthalene in $CDCl_3$.

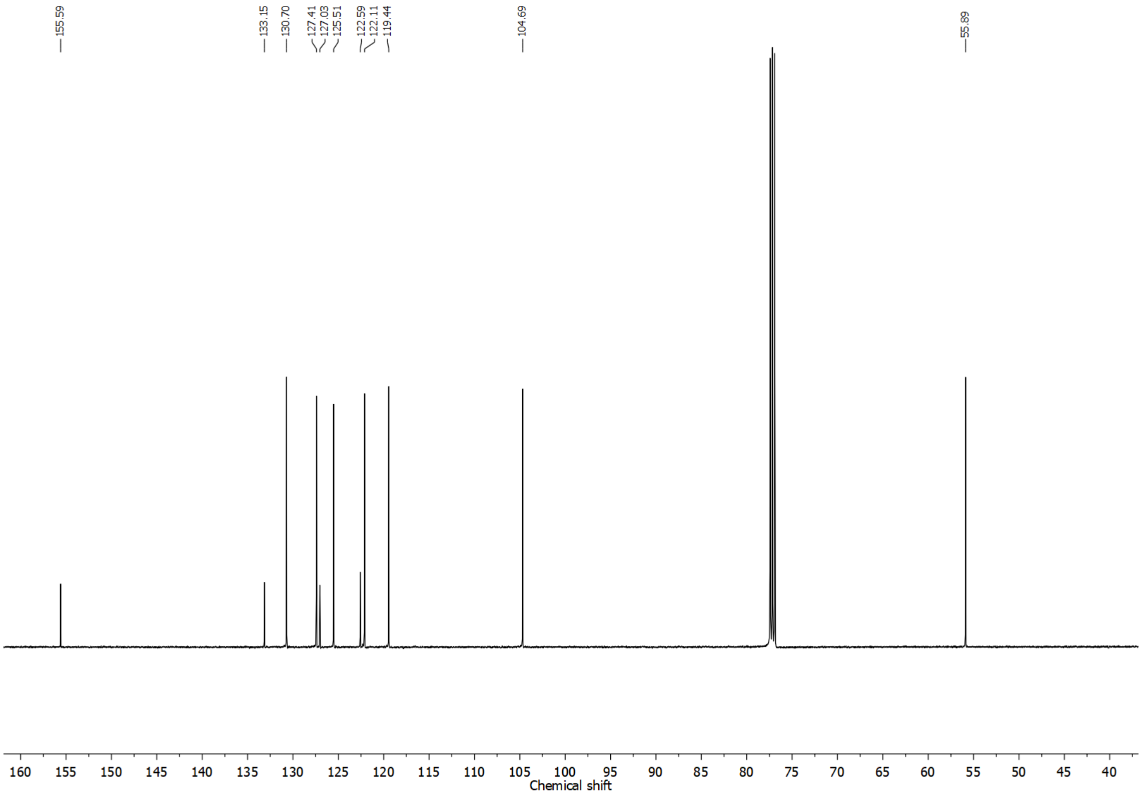

Figure S9. ^{13}C NMR spectrum (125 MHz) of 1-bromo-5-methoxynaphthalene in $CDCl_3$.

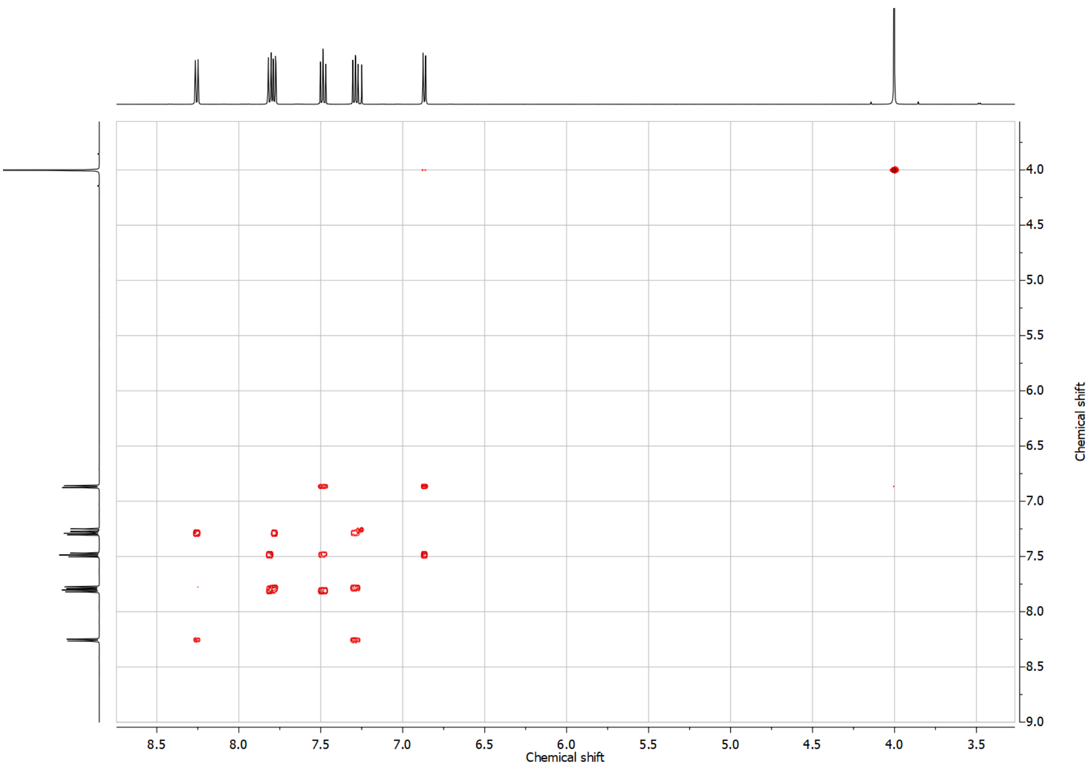

Figure S10. H,H-COSY spectrum (500 MHz) of 1-bromo-5-methoxynaphthalene in $CDCl_3$.

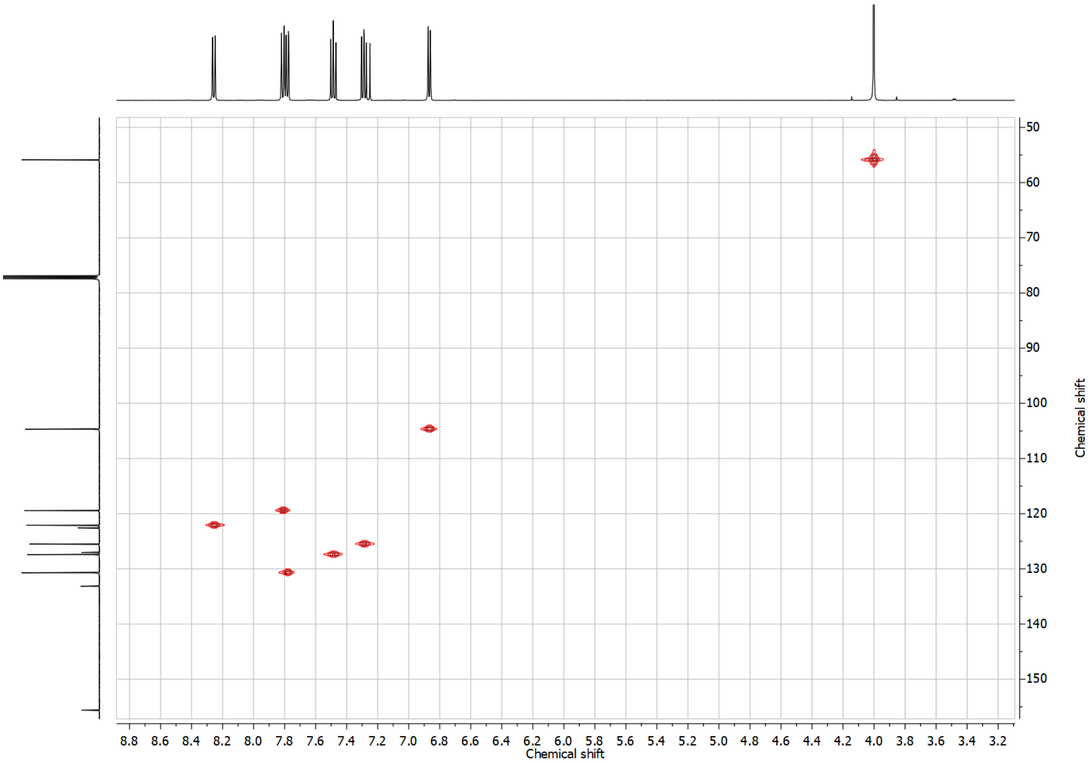

Figure S11. HSQC NMR spectrum (500 MHz) of 1-bromo-5-methoxynaphthalene in $CDCl_3$.

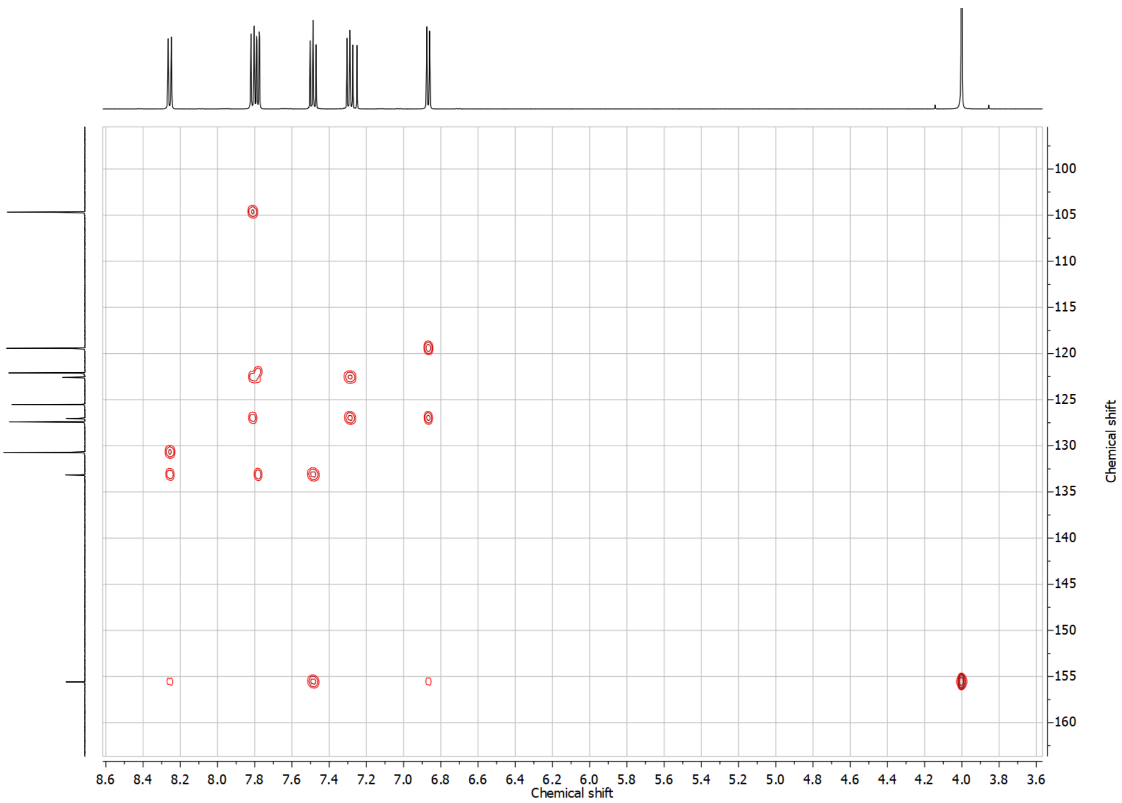

Figure S12. HMBC NMR spectrum (500 MHz) of 1-bromo-5-methoxynaphthalene in $CDCl_3$.

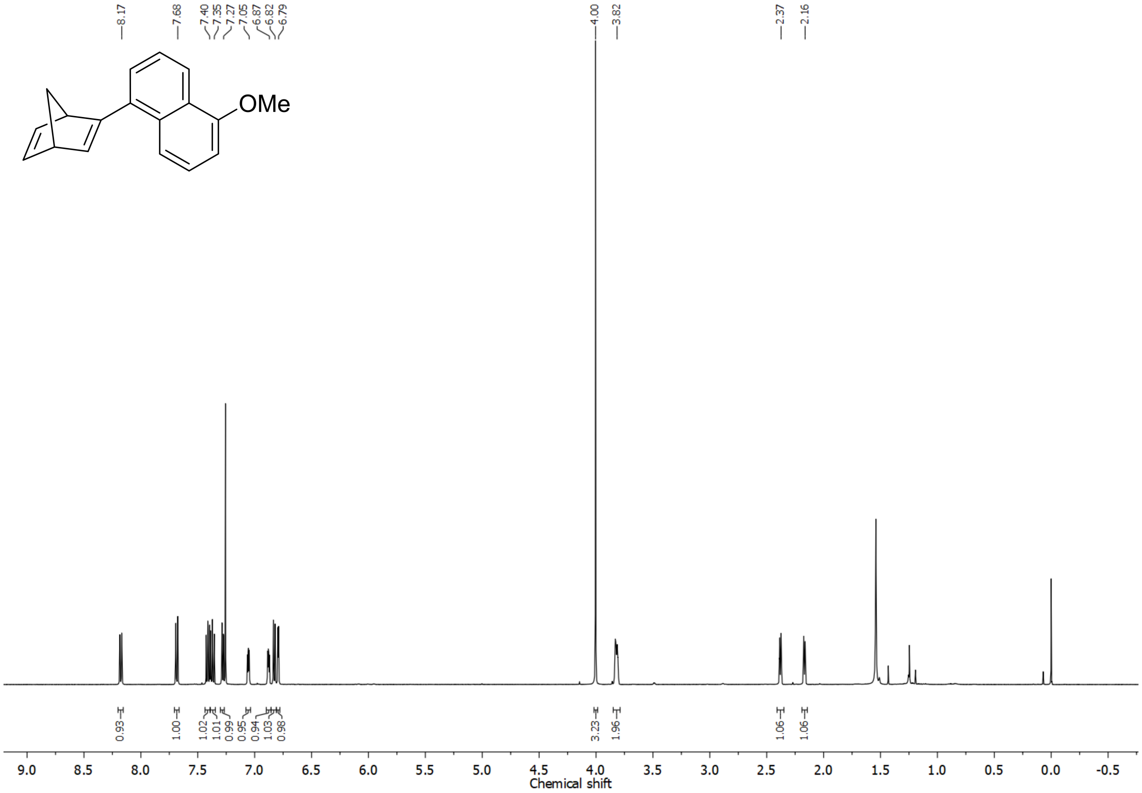

Figure S13. ^1H NMR spectrum (500 MHz) of **1d** in $CDCl_3$.

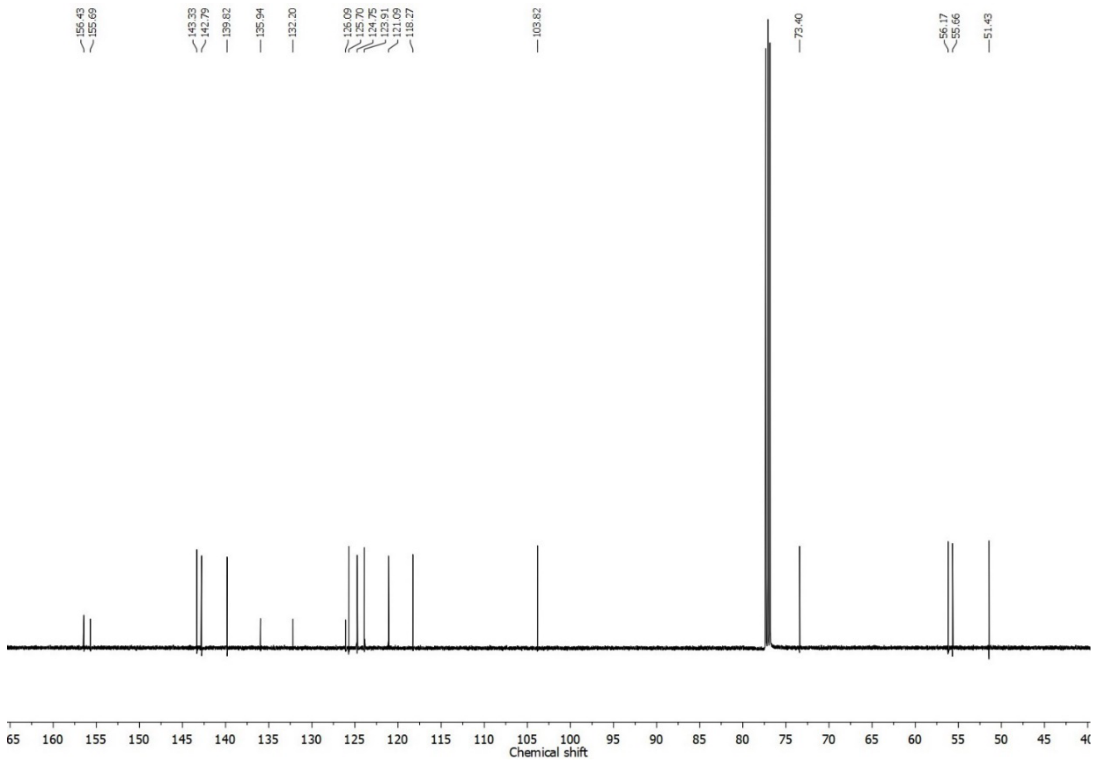

Figure S14. ^{13}C NMR spectrum (125 MHz) of **1d** in $CDCl_3$.

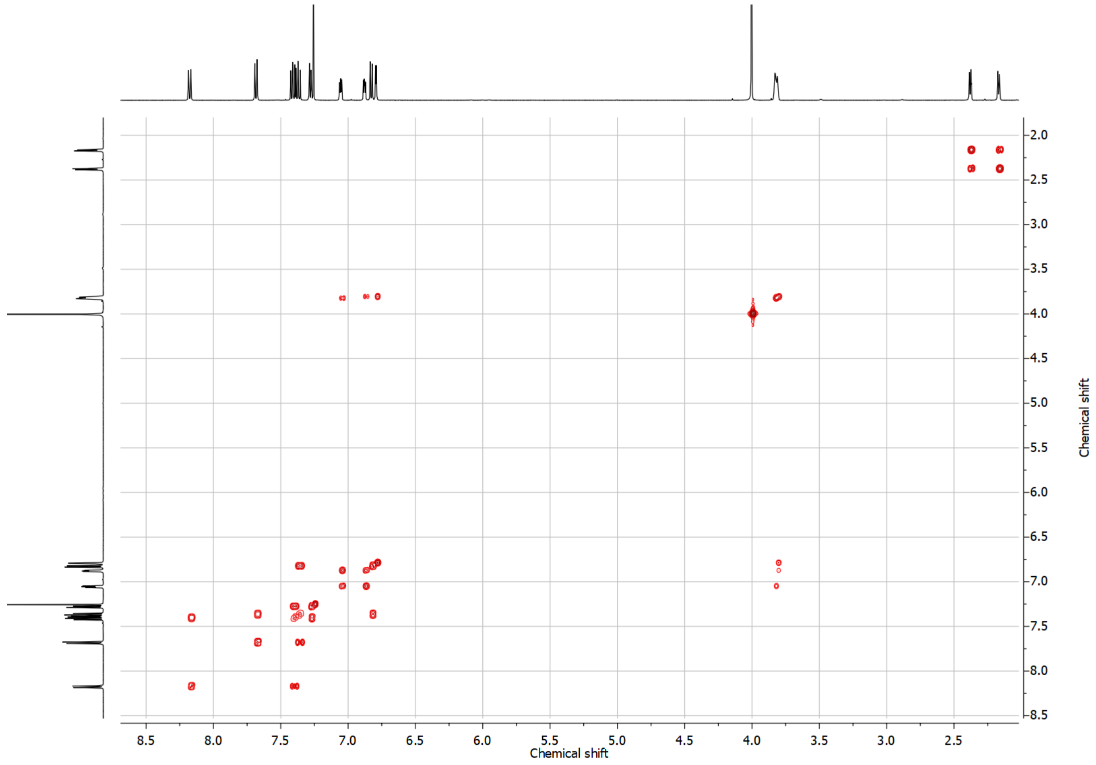

Figure S15. H,H-COSY spectrum (500 MHz) of **1d** in $CDCl_3$.

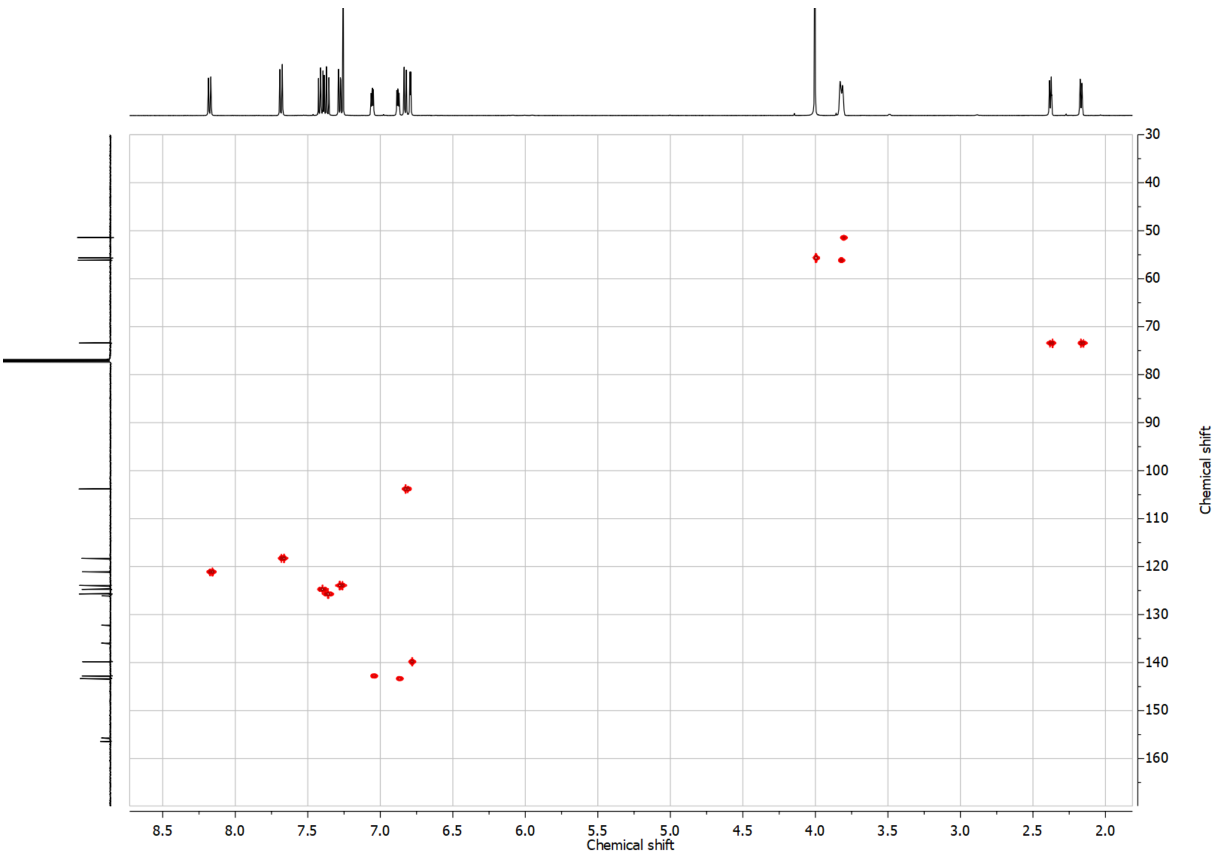

Figure S16. HSQC NMR spectrum (500 MHz) of **1d** in $CDCl_3$.

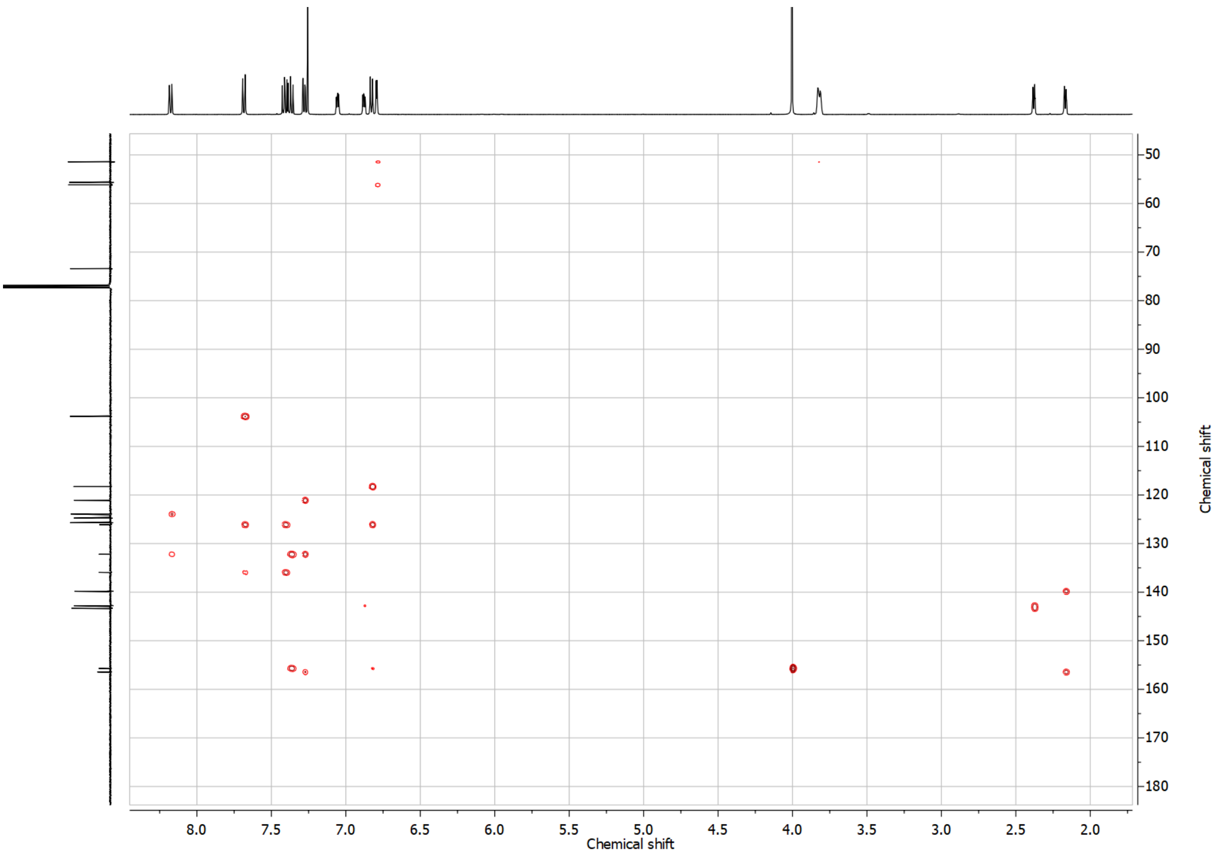

Figure S17. HMBC NMR spectrum (500 MHz) of **1d** in $CDCl_3$.

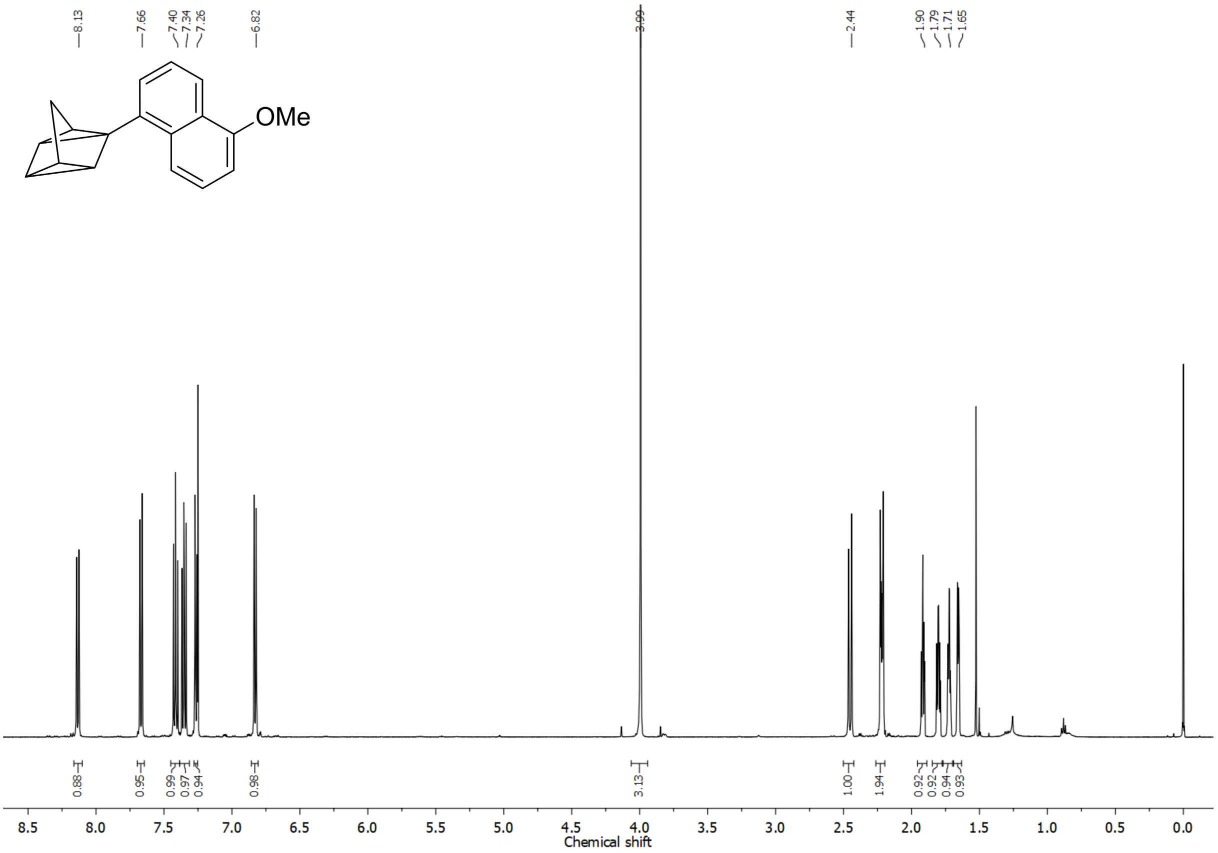

Figure S18. ^{1}H NMR spectrum (500 MHz) of **2d** in $CDCl_3$.

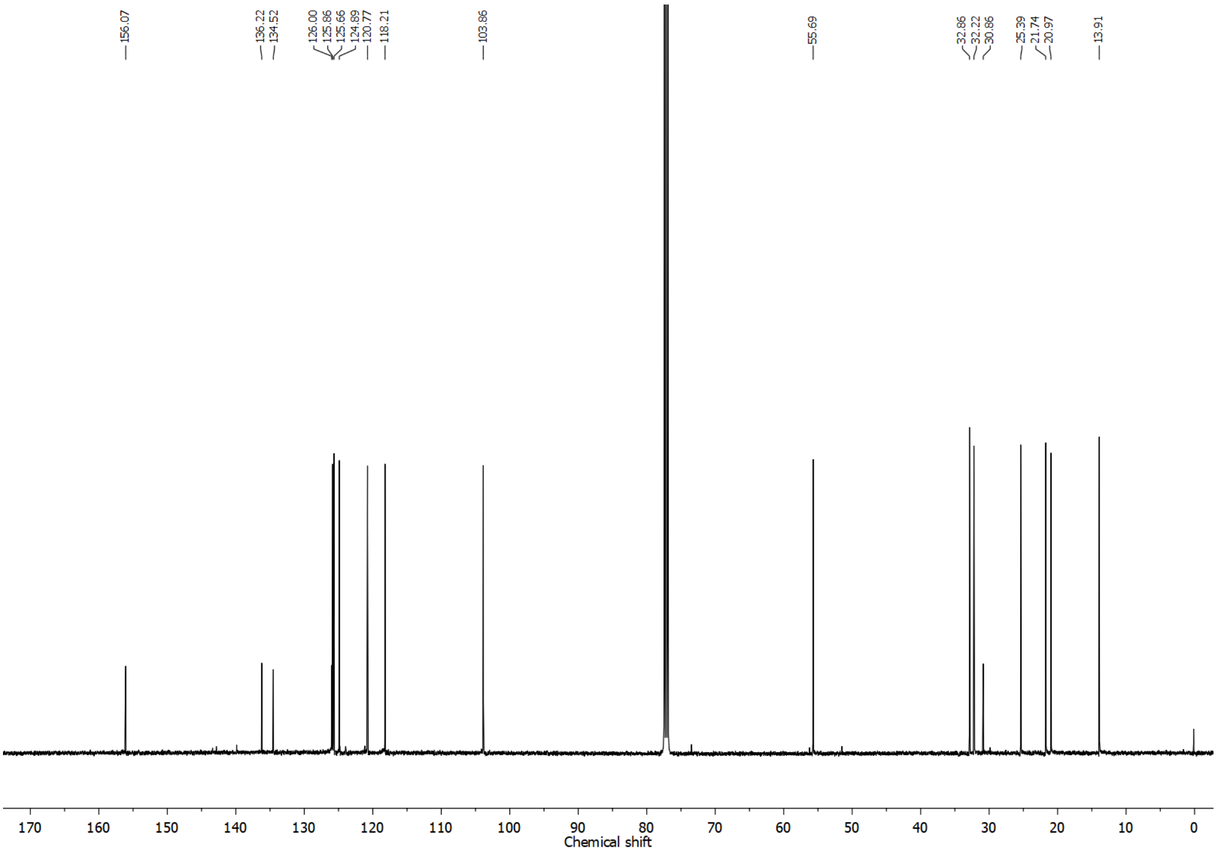

Figure S19. ^{13}C NMR spectrum (125 MHz) of **2d** in $CDCl_3$.

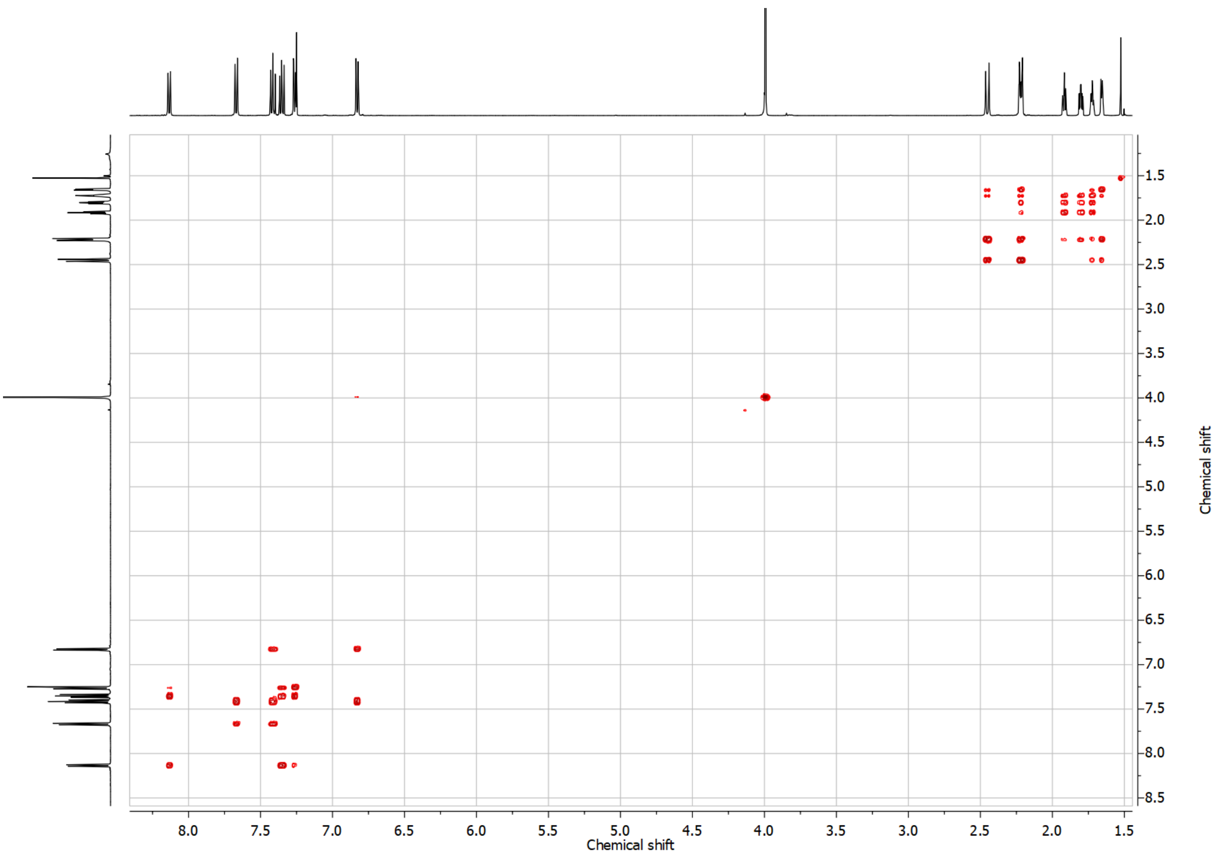

Figure S20. H,H-COSY spectrum (500 MHz) of **2d** in $CDCl_3$.

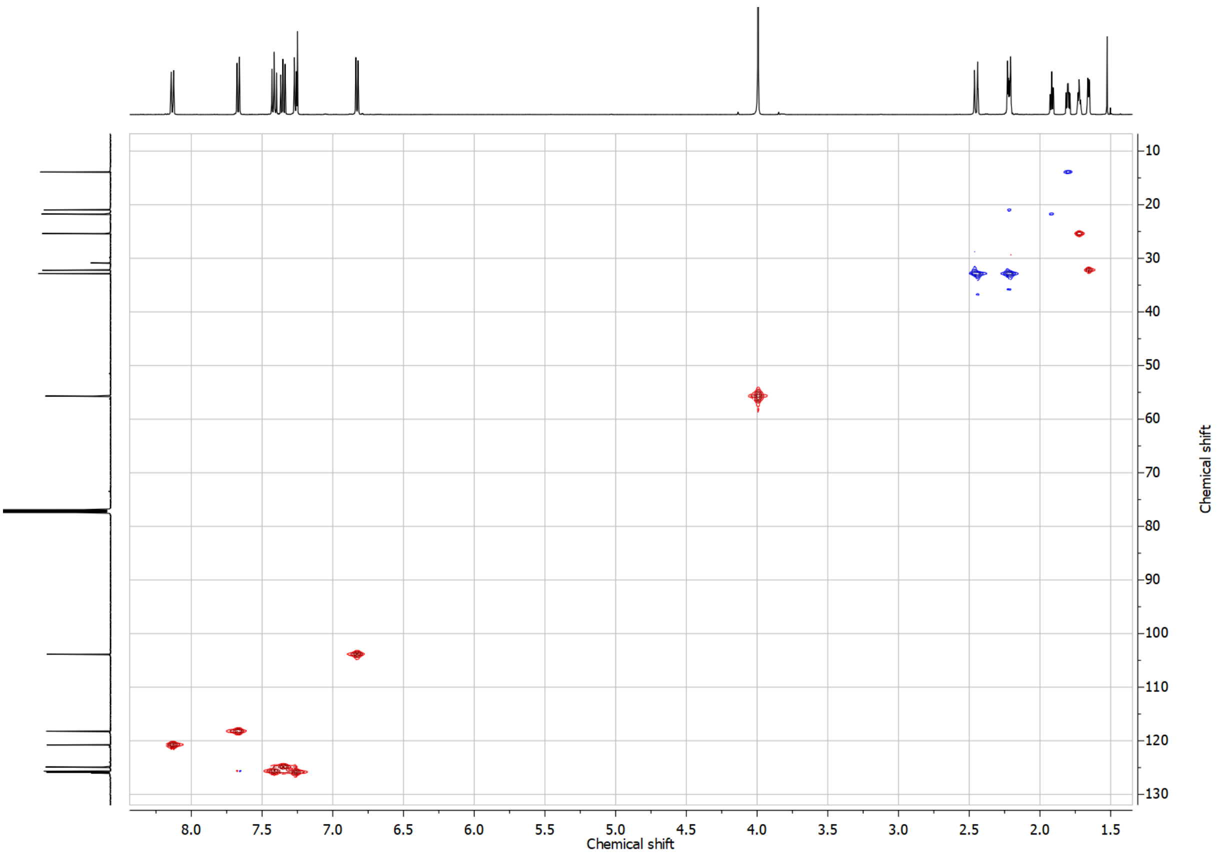

Figure S21. HSQC NMR spectrum (500 MHz) of **2d** in $CDCl_3$.

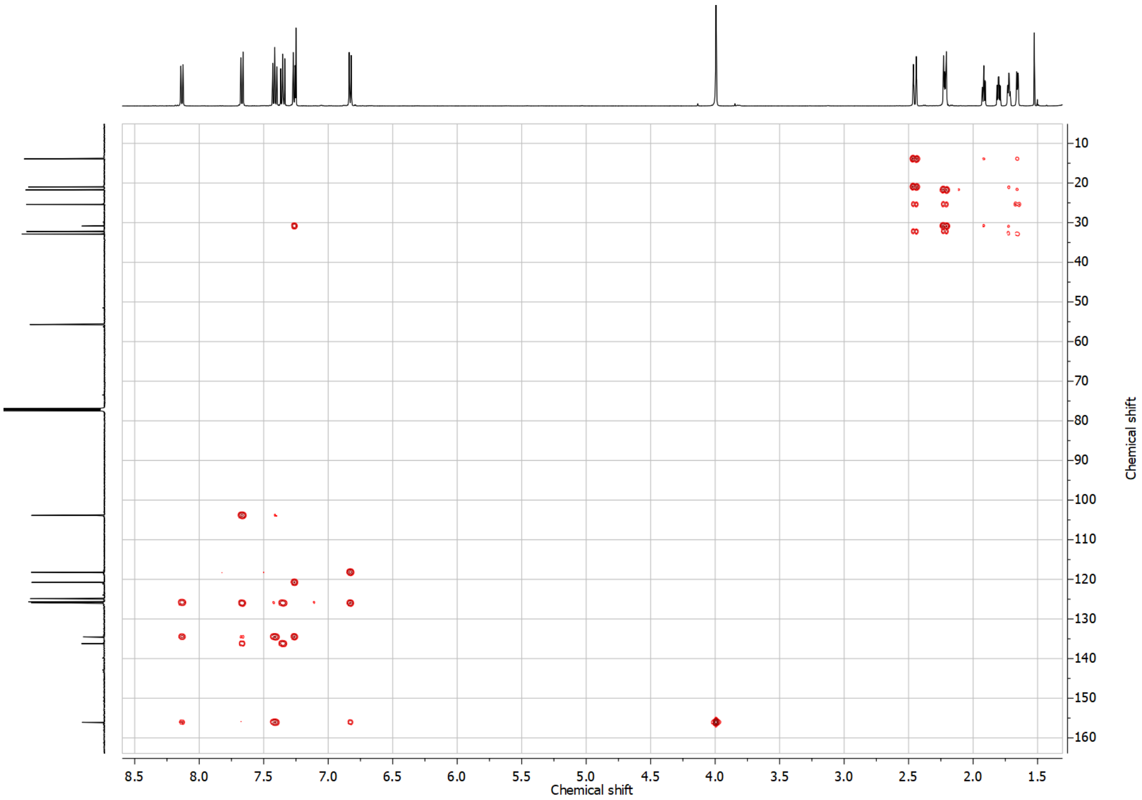

Figure S22. HMBC NMR spectrum (500 MHz) of **2d** in $CDCl_3$.

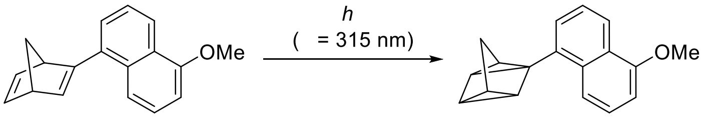

1d **2d**

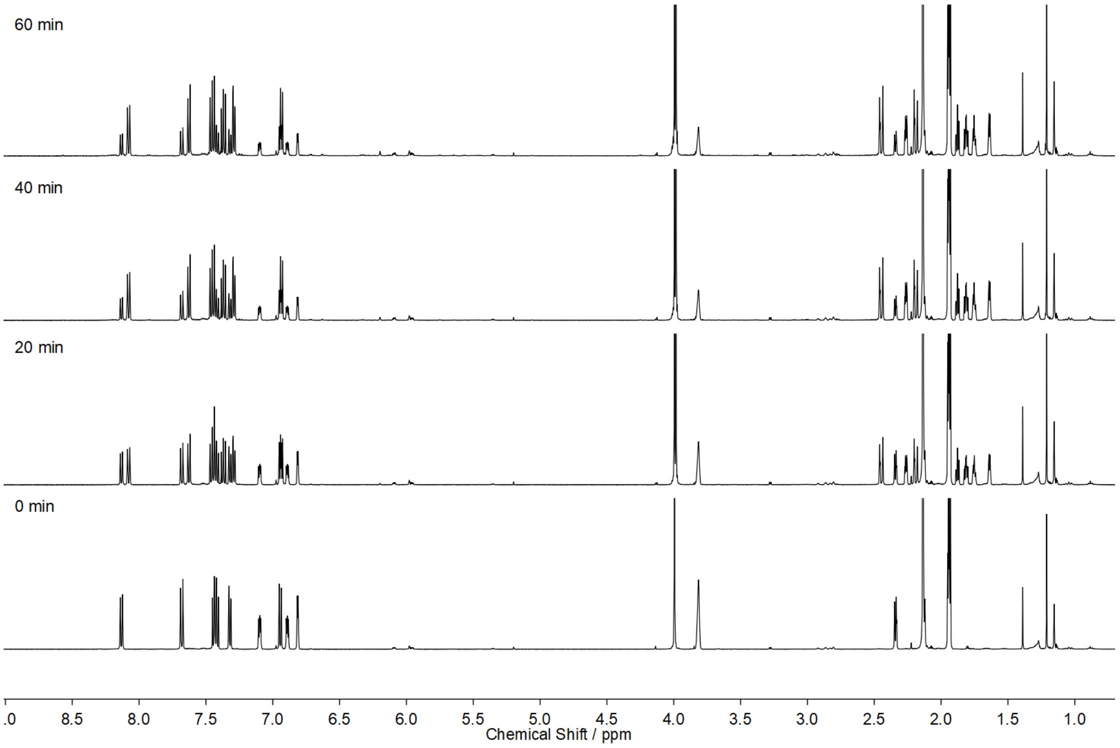

Figure S23. ^{1}H NMR spectroscopic tracking of the cycloaddition reaction of **1d** by irradiation with LUMOS 43 (λ_{ex} = 315 nm)

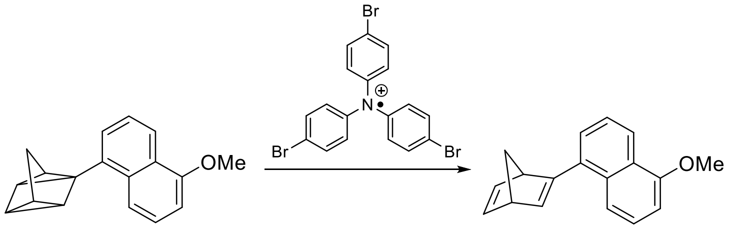

2d **1d**

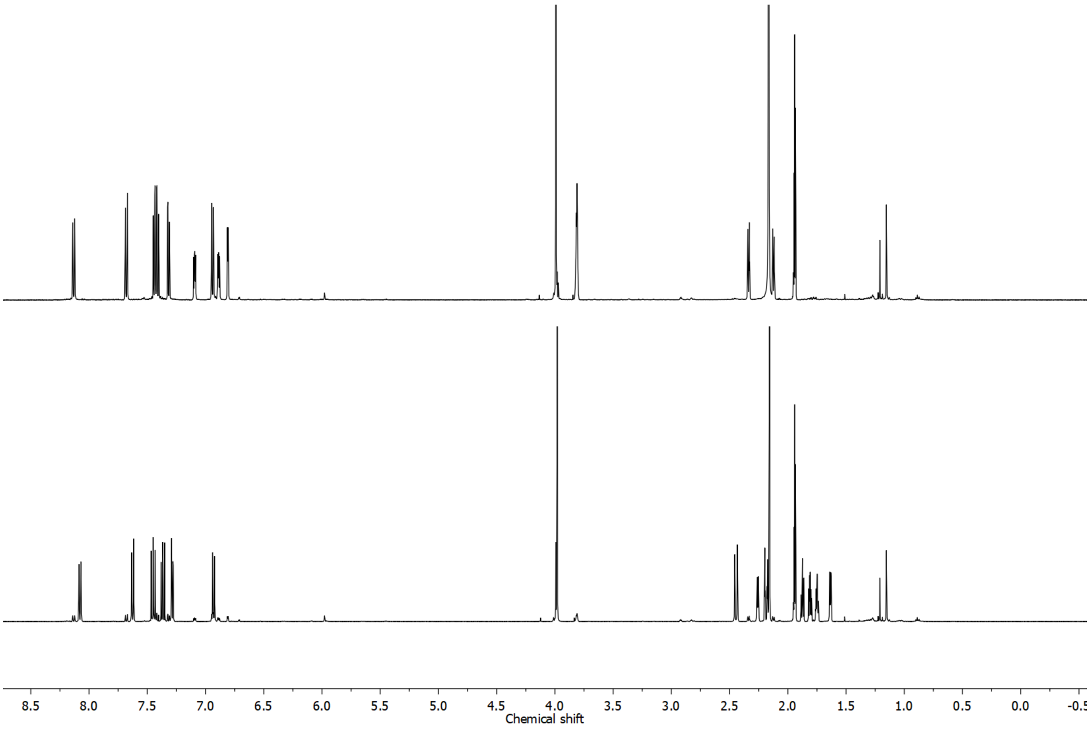

Figure S24. ^{1}H NMR spectra from **2d** after irradiation of **1d** (GP B, bottom spectrum) and after addition of the magic blue (1 mol%, above spectrum)

12. In-situ NMR spectroscopy

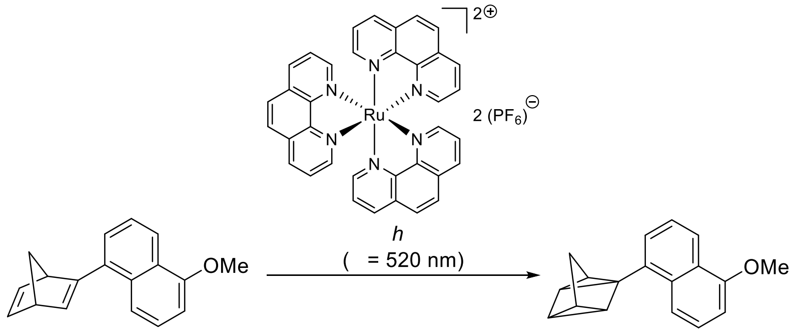

1d **2d**

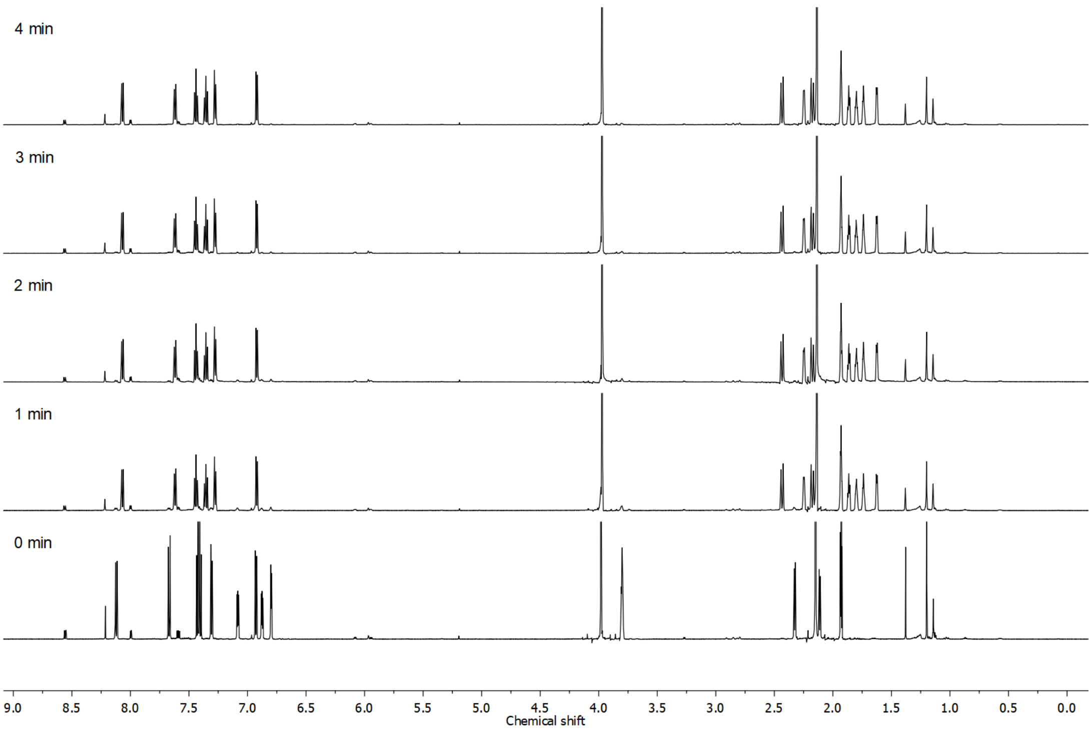

Figure S25. Selected in-situ ^{1}H NMR spectra (600 MHz) of **1d** (60 mM) with $(Ru(phen)_3(PF_6)_2$ (1 mol%) in MeCN-d_3 after variable irradiation time λ_{ex} = 520 nm. In-situ NMR spectra were recorded every minute, only selected are shown.

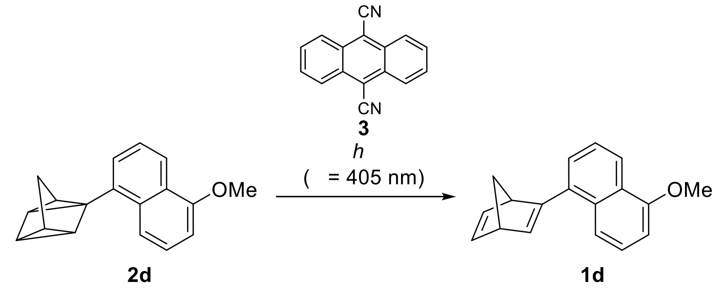

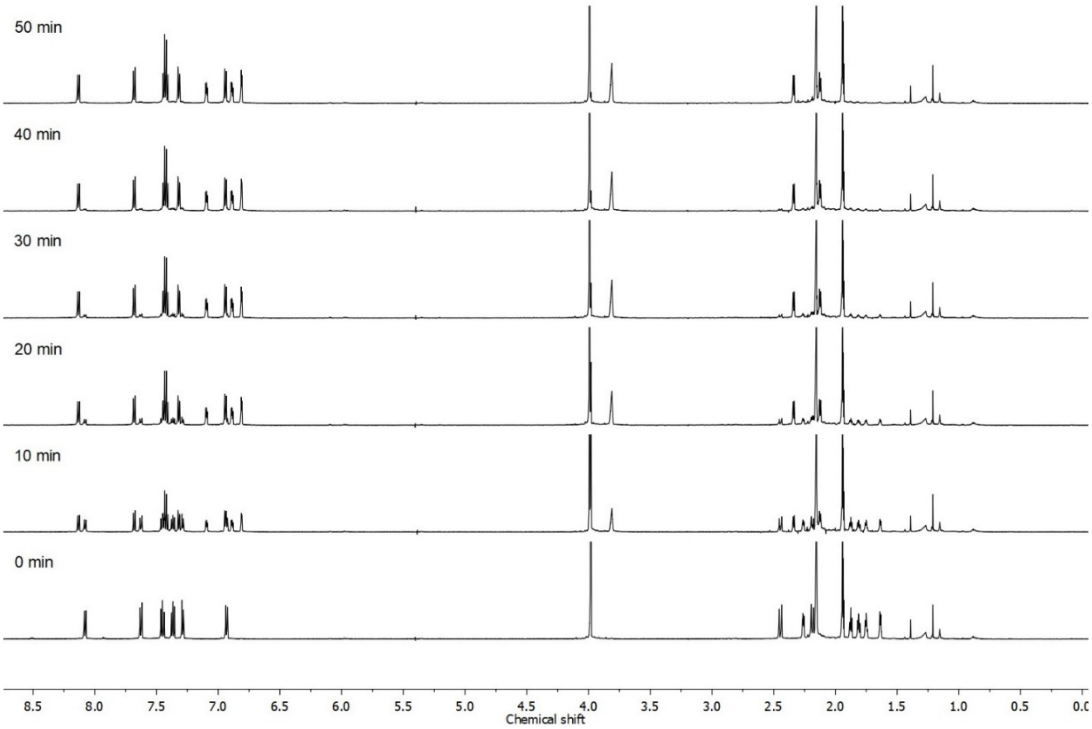

Figure S26. Selected in-situ ^{1}H NMR spectra (600 MHz) of **2d** (60 mM) with **3** (1 mol%) in MeCN-d_3 after variable irradiation time λ_{ex} = 405 nm. In-situ NMR spectra were recorded every minute, only selected are shown.

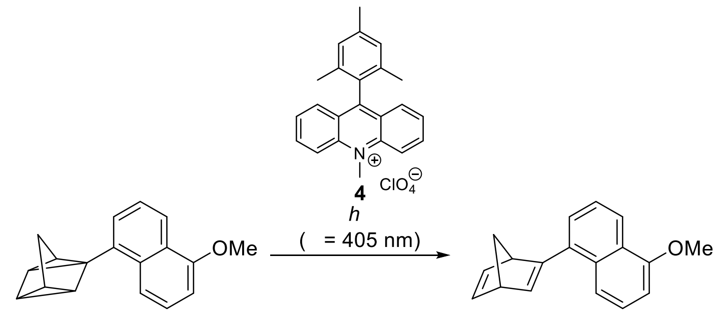

2d **1d**

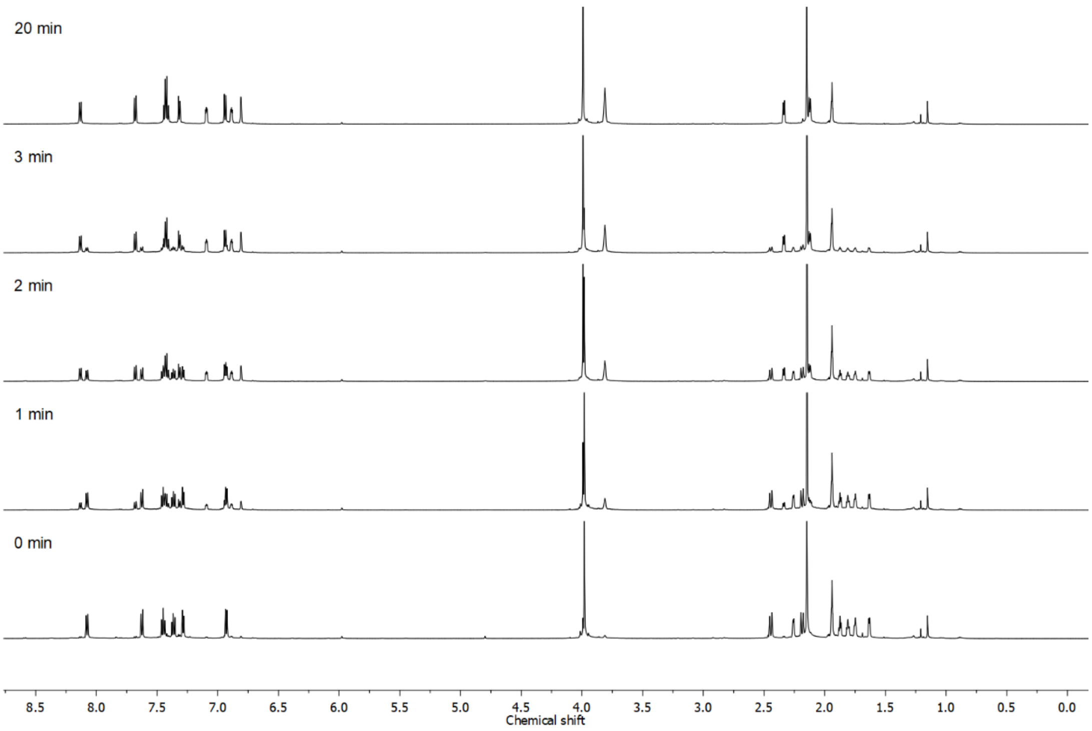

Figure S27. Selected in-situ ^{1}H NMR spectra (600 MHz) of **2d** (60 mM) with **4** (1 mol%) in MeCN-d_3 after variable irradiation time λ_{ex} = 405 nm. In-situ NMR spectra were recorded every minute, only selected are shown.

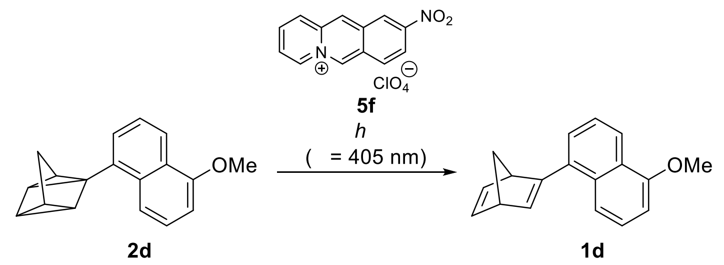

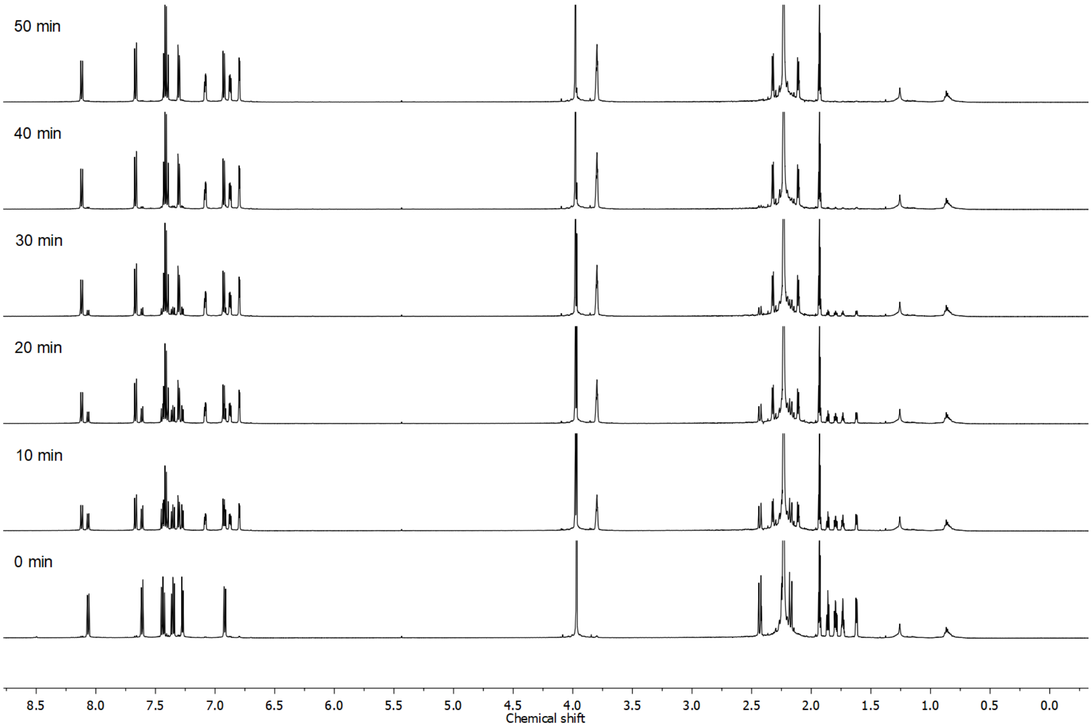

Figure S28. Selected in-situ ^{1}H NMR spectra (600 MHz) of **2d** (60 mM) with **5f** (1 mol%) in MeCN-d_3 after variable irradiation time λ_{ex} = 405 nm. In-situ NMR spectra were recorded every minute, only selected are shown.

13. Cyclovoltammetric analysis

The cyclovoltammetric measurements were performed in anhydrous DMF with tetrabutylammonium hexafluorophosphate (0.1 M) as the supporting electrolyte E_{pc} = −0.85 V_{FC} = −0.16 V_{NHE}, E_{pa} = 0.32 V_{FC} (Figure 29A).[47]

The reduction potential in the excited state, E_{red}^{*}, of **5f** was calculated according to equation 5.[39]

$$E_{\text{red}}^{\ *} = E_{\text{red}} + E_{0-0} \qquad \text{(eq. 5)}$$

In equation 5, E_{red} corresponds to the cathodic peak E_{pc} (Figure 29A). All values were converted to the ones relative to NHE.[48] $E_{0–0}$ is the energy of the 0–0 transition between ground and excited state. The $E_{0–0}$ value was calculated with eq. 6. In equation 6, h corresponds to the Planck constant and c to the speed of light. $\lambda_{0–0}$ = 438 nm is the wavelength at the intersection between the normalized absorption and emission wavelength (Figure 29B).

$$E_{0-0} = \frac{hc}{\lambda_{0-0}} \qquad \text{(eq. 6)}$$

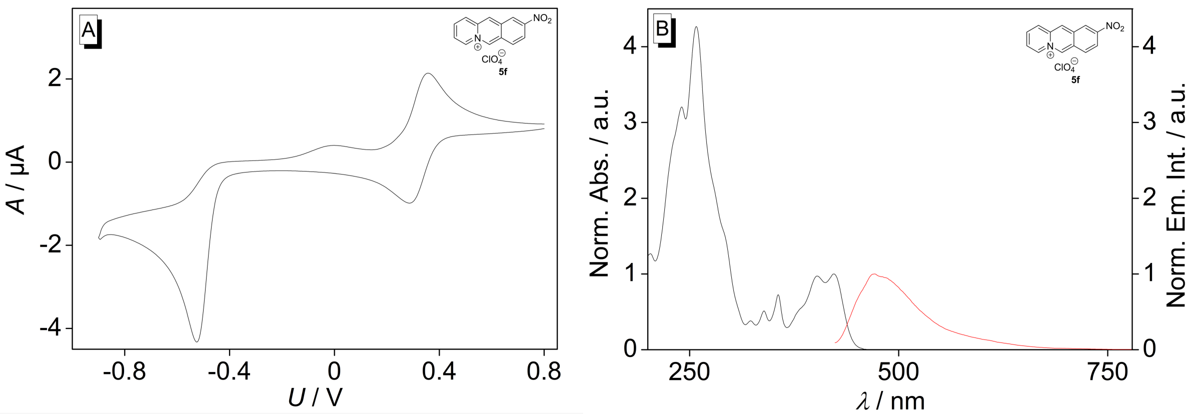

Figure S29. Cyclovoltammogram of **5f** in DMF (c = 1 mM) with ferrocene as internal standard (A) and absorption (—) and emission (—) spectrum of **5f** (B).

14. References

[11] T. J. B. Zähringer, N. P. Lopez, R. Schulte, M. Schmitz, H. Ihmels, C. Kerzig, *Angew. Chem. Int. Ed.* **2024**, *64*, e202414733.

[30] a) R. Schulte, S. Afflerbach, T. Paululat, H. Ihmels, *Angew. Chem. Int. Ed.* **2023**, *62*, e202309544; b) R. Schulte, H. Ihmels, *Beilstein J. Org. Chem.* **2022**, *18*, 368–373; d) R. Schulte, D. Schade, T. Paululat, T. J. B. Zähringer, C. Kerzig, H. Ihmels, *Beilstein J. Org. Chem.* **2024**, *20*, 3061–3068.

[32] T. Paululat, M. Rabe, D. V. Berdnikova, *J. Magn. Reson.* **2021**, *327*, 106990.

[36] a) A. P. Krapcho, S. A. Cadamuro, L. Macnee, *Arkivoc* **2007**, *ix*, 28–44; b) S. A. Stratford, M. Arhangelskis, D.-K. Bučar, W. Jones, *CrystEngComm* **2014**, *16*, 10830–10836; c) C. K. Bradsher, J. C. Parham, *J. Heterocycl. Chem.* **1964**, *1*, 30–33; d) W. G. Earley, J. A. Dority Jr., V. Kumar, J. P. Mallamo, *Heterocycles* **1995**, *41*, 309–314.

[39] Y. Wu, D. Kim, T. S. Teets, *Synlett* **2021**, *33*, 1154–1179.

[44] W. J. Olivier, P. Błyszczyk, E. M. Arpa, K. Hitoshio, M. Gomez-Mendoza, V. de la Peña O'Shea, I. Marchand, T. Poisson, A. Ruffoni, D. Leonori, *Science* **2025**, 387, 1167–1174.

[45] C. G. Hatchard, C. A. Parker, *Proc. R. Soc. Lond. Ser. A* **1956**, 235, 518–536.

[46] K. Stranius, K. Börjesson, *Sci. Rep.* **2017**, 7, 41145.

[47] R. Mitzner, J. Bendig, R. Ziebig, F. Graichen, D. Kreysig, F. Pragst, *J. Prakt. Chem.* **1985**, *327*, 241–250.

[48] a) N. G. Connelly, W. E. Geiger, *Chem. Rev.* **1996**, *96*, 877–910; b) V. V. Pavlishchuk, A. W. Addison, *Inorganica Chim. Acta* **2000**, *298*, 97–102.
